# ERF9 of *Poncirus trifoliata* (L.) Raf. undergoes feedback regulation by ethylene and modulates cold tolerance via regulating a *glutathione S‐transferase U17* gene

**DOI:** 10.1111/pbi.13705

**Published:** 2021-09-29

**Authors:** Yang Zhang, Ruhong Ming, Madiha Khan, Yue Wang, Bachar Dahro, Wei Xiao, Chunlong Li, Ji‐Hong Liu

**Affiliations:** ^1^ Key Laboratory of Horticultural Plant Biology College of Horticulture and Forestry Sciences Huazhong Agricultural University Wuhan China

**Keywords:** Trifoliata orange, ERF, cold tolerance, ROS homeostasis, ethylene biosynthesis, *glutathione S‐transferase*, *ACC synthase*

## Abstract

Plant ethylene‐responsive factors (ERFs) play essential roles in cold stress response, but the molecular mechanisms underlying this process remain poorly understood. In this study, we characterized *PtrERF9* from trifoliate orange (*Poncirus trifoliata* (L.) Raf.), a cold‐hardy plant. *PtrERF9* was up‐regulated by cold in an ethylene‐dependent manner. Overexpression of *PtrERF9* conferred prominently enhanced freezing tolerance, which was drastically impaired when *PtrERF9* was knocked down by virus‐induced gene silencing. Global transcriptome profiling indicated that silencing of *PtrERF9* resulted in substantial transcriptional reprogramming of stress‐responsive genes involved in different biological processes. PtrERF9 was further verified to directly and specifically bind with the promoters of *glutathione S‐transferase U17* (*PtrGSTU17*) and *ACC synthase1* (*PtrACS1*). Consistently, *PtrERF9*‐overexpressing plants had higher levels of *PtrGSTU17* transcript and GST activity, but accumulated less ROS, whereas the silenced plants showed the opposite changes. Meanwhile, knockdown of *PtrERF9* decreased *PtrACS1* expression, ACS activity and ACC content. However, overexpression of *PtrERF9* in lemon, a cold‐sensitive species, caused negligible alterations of ethylene biosynthesis, which was attributed to perturbed interaction between PtrERF9, along with lemon homologue ClERF9, and the promoter of lemon *ACS1* gene (*ClACS1*) due to mutation of the *cis*‐acting element. Taken together, these results indicate that *PtrERF9* acts downstream of ethylene signalling and functions positively in cold tolerance via modulation of ROS homeostasis by regulating *PtrGSTU17*. In addition, PtrERF9 regulates ethylene biosynthesis by activating *PtrACS1* gene, forming a feedback regulation loop to reinforce the transcriptional regulation of its target genes, which may contribute to the elite cold tolerance of *Poncirus trifoliata*.

## Introduction

Citrus is an economically important fruit crop in the world and occupies a key position in international fruit trade. Being sessile organism, citrus production is constantly subjected to a myriad of environment stresses, among which low temperature is a major limiting factor for plant growth, development, geographical distribution and productivity (Ding *et al*., 2019; Zhu, 2016). Thus, improving the cold resistance of citrus is regarded as an important breeding programme. Trifoliate orange (*Poncirus trifoliata* (L.) Raf.), closely related to citrus, exhibits desirable resistance to extremely low temperature when fully acclimated (Peng *et al*., 2014). It is usually used as a citrus rootstock to incorporate its elite tributes. In addition, it is widely accepted that trifoliate orange is a good germplasm source for exploring cold‐responsive genes that can be employed for genetic engineering for manipulating cold tolerance. To this end, great efforts have been devoted to elucidating molecular mechanisms underlying the cold tolerance of trifoliate orange and to identifying valuable genes that are tightly involved in this process and play key roles in modulating the cold response.

Plant perceived stress signals through various signalling pathways mediated by different signalling molecules, including phytohormones, reactive oxygen species (ROS) and Ca^2+^ (Guo *et al*., 2018; Hu *et al*., 2017; Peng *et al*., 2017). These signalling pathways play crucial roles in orchestration of stress signal perception, signal transduction and magnification, and finally trigger a large spectrum of stress responses at physiological, biochemical, metabolic and molecular levels (Ding *et al*., 2020; Zhang *et al*., 2019a). Among the phytohormones, ethylene has been increasingly regarded as an important member helping the plants adapt to adverse environmental stresses. This argument is supported by the findings that ethylene synthesis is prominently altered by diverse stresses, such as drought, cold, wounding and pathogen attack (Wang *et al*., 2021; Müller and Munné‐Bosch, 2015). Interestingly, ethylene has been shown to act as either a positive or negative regulator of cold stress response. Some studies showed that increased endogenous ethylene level was accompanied by enhanced cold tolerance of grapevine and apple (Sun *et al*., 2016; Wang *et al*., 2021). By contrast, ethylene levels were negatively correlated with freezing tolerance of *Arabidopsis thaliana* and *Medicago truncatula* (Shi *et al*., 2012; Zhao *et al*., 2014). In addition, treatment with 1‐methylcyclopropene (1‐MCP), an inhibitor of ethylene signal perception, increased the chilling injury of tomato fruit (Zhao *et al*., 2009). Despite the contrasting conclusions obtained in different plant species, these findings suggested that ethylene may be tightly implicated in cold tolerance. However, the molecular network pertinent to ethylene biosynthesis in response to cold stress remains largely uncharacterized in most plants.

Plants have evolved a sophisticated adaptation mechanism so as to maintain growth and development and to survive under the harsh environmental cues. One of the mechanisms is the transcriptional reprogramming of a vast number of stress‐responsive molecular modules composed of transcription factors (TFs) and their corresponding target genes. The TFs serve as the indispensable links connecting the external environmental stimuli by relaying the stress signal to the downstream gene expression. The APETALA2/ethylene‐responsive factors (AP2/ERFs) superfamily is one of the largest plant‐specific TF families and functions as the major regulatory factors for ethylene signalling pathway in plant stress responses. The AP2/ERF proteins, defined by containing one or two conserved AP2 domains, are divided into three separate subfamilies, ERF, AP2 and RAV (Licausi *et al*., 2013). Accumulating evidence has demonstrated that the AP2/ERF TFs are involved in a wide range of environmental stresses, including drought (Zhao *et al*., 2020, 2020,2020, 2020), heavy metal (Lin *et al*., 2017), salinity (Schmidt *et al*., 2013), high light (Vogel *et al*., 2014) and cold (Wang *et al*., 2019; Zhuo *et al*., 2018), in a variety of plant species. However, it is worth mentioning that the roles of most ERF members in cold tolerance remain far from being clearly characterized. In addition, the target genes by which the ERFs regulate to orchestrate the cold tolerance remain largely ambiguous, which hampers our integrative understanding of the cold stress response at molecular levels.

Cold stress influences plant growth by causing several negative effects. One of them is increased production of ROS, which, at high concentrations, result in membrane lipid peroxidation, protein and nucleic acids oxidation, leading to cell structure damages or ultimately cell death (Considine and Foyer, 2014). Plants have developed non‐enzymatic and enzymatic antioxidant defence systems for removing excessive ROS produced under cold conditions. Glutathione S‐transferases (GST) is one of the enzymes that are mobilized by plants to scavenge ROS (Cui *et al*., 2019). Earlier studies have shown that increased GST activity or expression conferred abiotic stress tolerance, whereas loss‐of‐function of *GST* in rice mutant *osgst4* was found to exhibit reduced salt tolerance (Jha *et al*., 2011; Xu *et al*., 2018). The GST family is grouped into seven classes, tau (GSTU), phi (GSTF), lambda (GSTL), dehydroascorbate reductase (DHAR), zeta (GSTZ), theta (GSTT) and tetrachlorohydroquinone dehydrogenase (TCHQD), in which the first four GSTs are specific to plants (Rezaei *et al*., 2013). Currently, substantial evidence has suggested that GSTUs can scavenge H_2_O_2_ and O_2_^·−^ to modulate ROS homeostasis under various stresses (Horváth *et al*., 2020; Zhang *et al*., 2019b, 2019a). Although the GSTs have been well reported to participate in plant abiotic stress tolerance by elimination of ROS, the upstream regulatory proteins of the GSTs are still unclear. A raised question is whether the GSTs, in particular the plant‐specific ones, are regulated by the ERFs under cold stress.

In an earlier study, we carried out a global transcriptome analysis in *Poncirus trifoliata* subjected to cold treatment in order to identify cold‐inducible genes (Wang *et al*., 2015). A number of genes annotated as ERFs were induced to different extent by cold, and one of them (*Pt2g011090*), designated as *PtrERF9* in this study, was strongly induced. We thus hypothesize that PtrERF9 may act as a critical regulator of cold tolerance in trifoliate orange by modulation of pertinent metabolic pathways through its regulating target genes. Here, we demonstrated that *PtrERF9* plays a positive role in cold tolerance based on overexpression and virus‐induced gene silencing (VIGS) assays. By using RNA‐sequencing (RNA‐seq), we found that PtrERF9 may act to regulate a myriad of stress‐associated genes involved in various signalling and metabolic pathways. Furthermore, we demonstrate that PtrERF9 functions in cold tolerance by modulation of ROS homeostasis via directly targeting *PtrGSTU17*, a GSTU gene. Interestingly, ethylene participates in cold‐induced up‐regulation of *PtrERF9*, which was further demonstrated to regulate ethylene synthesis by interacting with the promoter of *PtrACS1*, but not with the promoter of *ClACS1* from lemon, implying that PtrERF9 undergoes a feedback regulation of ethylene to orchestrate cold stress response in trifoliate orange.

## Results

### Identification of *PtrERF9* based on a transcriptome dataset

**Figure 1:**
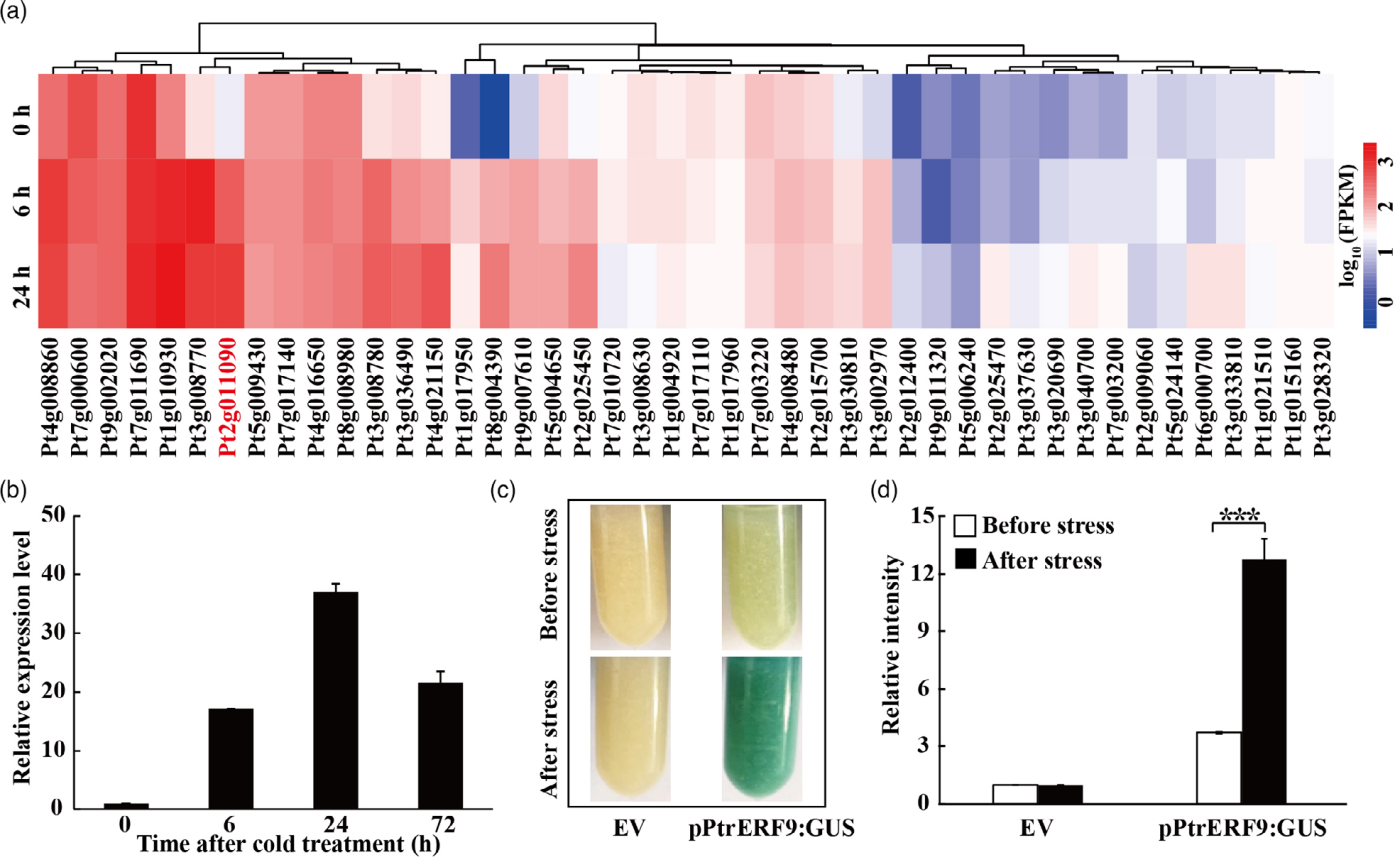
The expression of *ERF* genes analysis under cold treatment in *Poncirus trifoliata*. (a) Expression profiles of 44 *ERF* genes in trifoliate orange under cold stress. (b) Relative expression of *PtrERF9* was quantitated by qPCR in response to low temperature. Error bars represent ± SE (*n* = 3). (c, d) GUS staining (c) and relative GUS intensity (d) analysis in sweet orange callus transformation with empty vector (EV) and pPtrERF9: GUS in response to cold treatment. Error bars represent ± SE (*n* = 3). Asterisks indicate significant difference between the vectors under before and after the cold treatment (***P < 0.001).

The transcript profiles of ERF genes in cold‐treated trifoliate orange were examined using a heat map based on our previous RNA‐seq data (Wang *et al*., 2015). Among the ERF genes, *Pt2g011090* was induced to the greatest extent by cold (Figure 1a). A full‐length cDNA sequence of *Pt2g011090* was obtained by RT‐PCR from trifoliate orange. The 696‐bp ORF encodes a predicted protein of 231 amino acids (aa), with an estimated molecular mass of 24.85 kDa and an isoelectric point of 9.15. Phylogenetic tree analysis revealed that *Pt2g011090* was most closely related to AtERF9 (Figure S1A & B), which allowed us to rename *Pt2g011090* as *PtrERF9* in this study. Protein structure analysis showed that PtrERF9 contained a conserved AP2 domain of 64 aa. Since the residues at the 14^th^ and 19^th^ positions in the AP2 domain were alanine (A) and aspartic acid (D), respectively (Figure S1C), PtrERF9 was categorized into the ERF subfamily.

### Cold induction of *PtrERF9* is dependent on ethylene

Expression pattern of *PtrERF9* under cold stress was checked by qPCR. *PtrERF9* transcript level was up‐regulated after low‐temperature treatment and peaked at 24 h (37‐fold of that at 0 h), followed by a steady decrease to 21‐fold of the initial level at the last time point (Figure 1b). In order to confirm this pattern, a transient expression assay was performed by expressing the GUS gene driven by the promoter of *PtrERF9* (pPtrERF9) in citrus callus. Histochemical staining showed that the GUS expression level in the calli expressing pPtrERF9:GUS construct was drastically increased following the cold treatment, justifying that *PtrERF9* is a cold‐responsive gene (Figures 1c and d).

**Figure 2:**
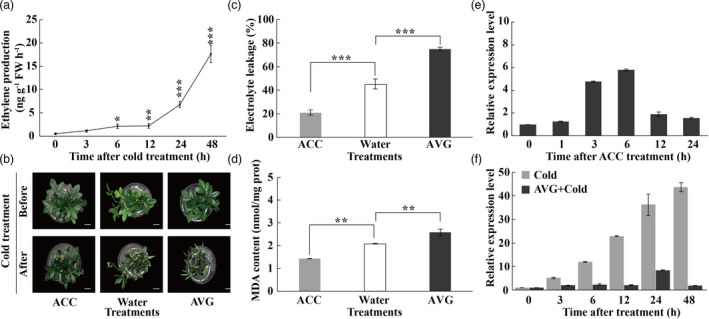
Ethylene enhanced trifoliate orange cold tolerance and *PtrERF9* expression was increased association with ethylene production under cold stress. (a) Cold induced ethylene production in trifoliate orange. (b) Phenotype of ACC‐, water‐ or AVG‐pretreated wild‐type plants in response to freezing treatment. Scale bars = 1 cm. (c, d) Electrolyte leakage (c) and MDA levels (d) in ACC‐, water‐, AVG‐pretreated wild‐type plants after cold treatment. Error bars indicate ± SE (*n* = 3). Asterisks indicate significant differences between different groups (**P < 0.01, ***P < 0.001). (e, f) The expression of *PtrERF9* under ACC treatment (e), cold and cold with AVG treatment (f) in trifoliate orange. Error bars indicate ± SE (*n* = 3).

Ethylene has been shown to play a vital role in plant response to various abiotic stresses, including cold (Sun *et al*., 2019). We also checked dynamic changes of ethylene in trifoliate orange exposed to cold condition. Ethylene production displayed a slow elevation at 12 h of cold treatment, followed by a sharp and steady increase to the highest level at 48 h (Figure 2a). Next, we explored whether ethylene contributed to cold tolerance by comparing plants treated with ACC and AVG, the precursor and inhibitor of ethylene biosynthesis, respectively, using water treatment as a control. No difference in plant morphology was observed between seedlings pretreated with ACC or AVG and water. However, upon exposure to cold treatment at freezing temperature, less serious necrotic phenotype, lower electrolyte leakage (EL) level and MDA content were noticed in the ACC‐treated seedlings, whereas the AVG‐treated seedlings plant exhibited more serious damages, concurrent with higher EL and MDA levels, in comparison with the water‐treated plants (Figure 2b–d). These results suggest that cold promoted ethylene production and that ethylene plays a positive role in cold tolerance of trifoliate orange.

It has been suggested that ERFs act downstream of ethylene signalling pathway to regulate abiotic stress response. This compels us to investigate how *PtrERF9* expression was influenced by ethylene under cold stress. To answer this question, we first determined *PtrERF9* expression in the presence of ACC. Interestingly, transcript level of *PtrERF9* was prominently increased by exogenous ACC treatment (Figure 2e). However, AVG application substantially compromised the cold induction of *PtrERF9* (Figure 2f). All of these data demonstrated that up‐regulation of *PtrERF9* by cold is dependent on synthesis ethylene.

### PtrERF9 is localized to the nucleus

**Figure 3:**
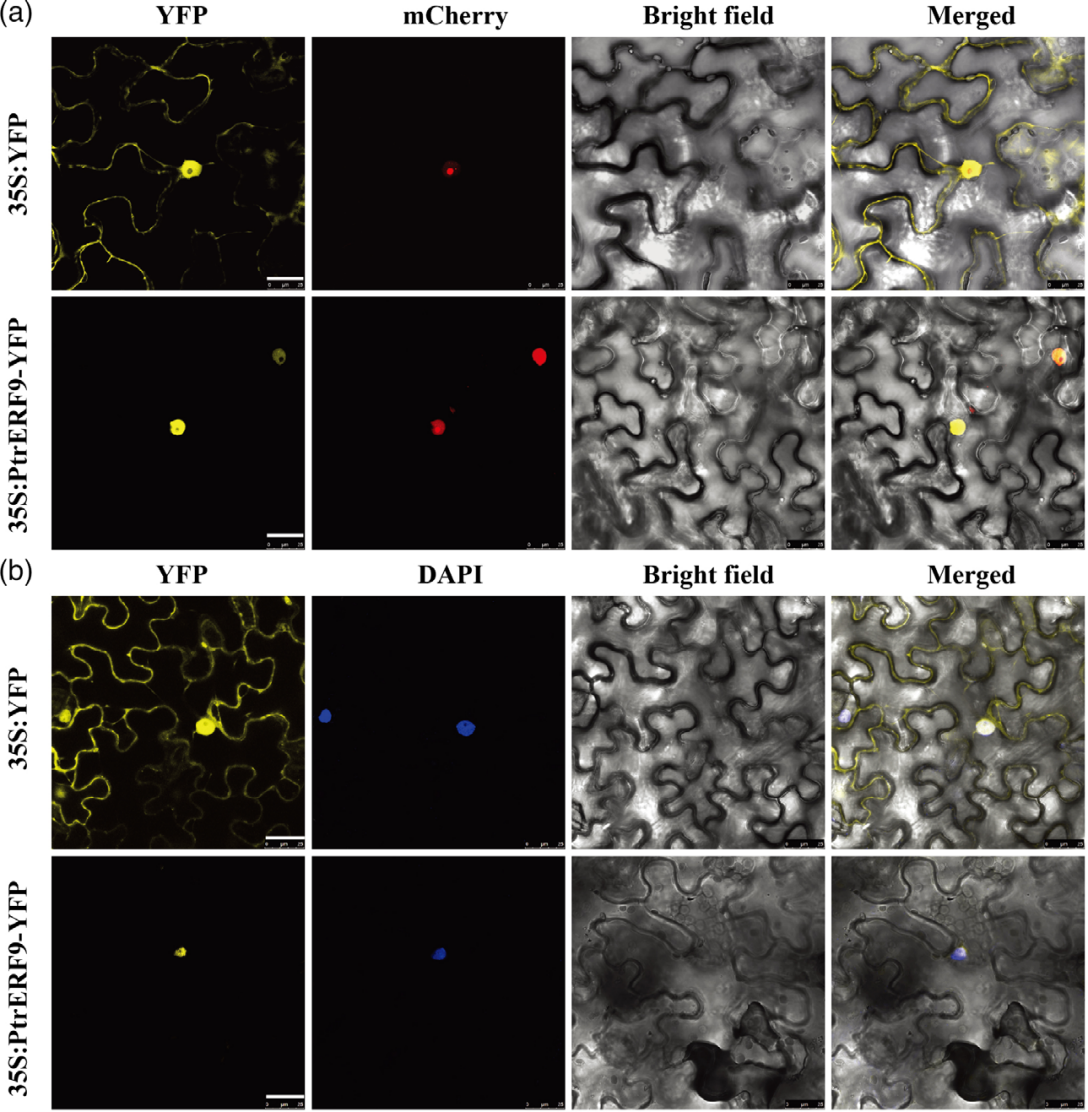
PtrERF9 is localized in the nucleus. (a) The fusion construct (35S: PtrERF9‐YFP) or an empty vector (35S: YFP) was co‐transformed with a nucleus marker gene *VirD2NLS* fused to mCherry in tobacco (*Nicotiana benthamiana*) leaves. Confocal microscopic images of the epidermal cells were taken under bright filed and yellow (for YFP), red (for mCherry) fluorescence signals. (b) DAPI staining, shown in blue, was used to stain the nucleus. The overlapped images are shown on the right. Scale bars = 25 µm.

To determine the subcellular location of PtrERF9, a construct was generated by fusing the *PtrERF9* cDNA to the *YFP* gene, driven by the CaMV *35S* promoter. Then, the 35S:PtrERF9‐YFP fusion construct or 35S:YFP empty vector was transiently expressed in *N. benthamiana* leaves via agroinfiltration. Confocal microscopic observation showed that the YFP signal was ubiquitously distributed in the cytoplasm and the nucleus of epidermal cells transformed with the control vector. However, when the fusion construct was examined, the YFP signal was detected exclusively in the nucleus, precisely co‐locating with the nuclear marker (mCherry, red fluorescence) or indicator (DAPI, blue fluorescence), implying that PtrERF9 is a nuclear protein (Figure 3).

### Overexpression of *PtrERF9* improves cold tolerance in transgenic plants

**Figure 4:**
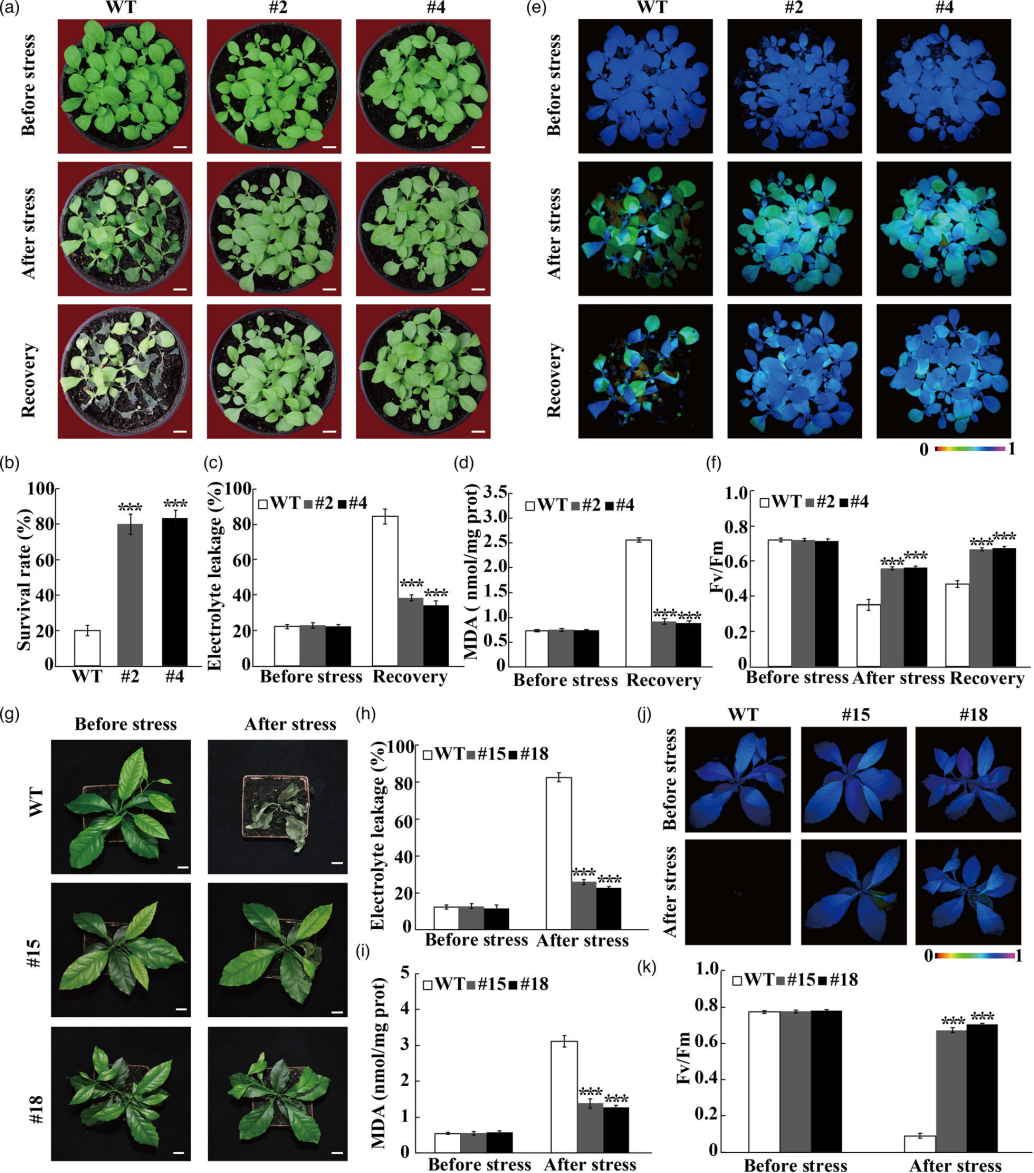
Overexpression of *PtrERF9* improved cold tolerance of transgenic plants. (a) Phenotypes of wild‐type (WT) and *PtrERF9* overexpression transgenic tobacco lines (#2 and #4) in response to cold treatment. Scale bars = 1 cm. (b) Survival rate of transgenic tobacco plants after the growth recovery for 3 d at ambient environment. (c, d) Electrolyte leakage (c), MDA content (d) of transgenic tobacco plants before and after the growth recovery for 3 d at 25 °C. (e, f) Chlorophyll fluorescence (e) and *F*_v_/*F*_m_ ratios (f) of transgenic tobacco plants before and after cold treatment and the growth recovery for 3 d at 25 °C. (g) Phenotypes of WT and *PtrERF9* overexpression transgenic lemon plants (#15 and #18) before and after cold treatment. Scale bars = 1 cm. (h‐k) Electrolyte leakage (h), MDA content (i), chlorophyll fluorescence (j) and *F*_v_/*F*_m_ (k) of WT and transgenic lemon before and after the cold treatment. Error bars represent ± SE (*n* = 3). Asterisks indicate significant difference between WT and the transgenic lines in response to cold treatment (***P < 0.001).

Since *PtrERF9* was dramatically induced by cold stress, we speculated that *PtrERF9* might play a pivotal role in regulation of cold tolerance. To verify this hypothesis, we generated *PtrERF9‐*overexpressing transgenic tobacco plants. We selected two independents lines (#2 and #4) with high transcript levels of *PtrERF9* for cold tolerance assay (Figure S2). No morphological differences were observed between the wild‐type (WT) and transgenic tobacco plants under normal growth conditions. After exposure to the cold condition at freezing temperature (−4°C for 6 h), the WT plants exhibited severe water‐soaking phenotype in comparison with the transgenic counterparts (Figure 4a). After recovery for 3 d at ambient temperature, 80%–85% of the transgenic plants survived, while the survival rate of WT was only 20% (Figure 4b). EL, MDA, chlorophyll fluorescence and Fv/Fm ratios between the WT and the transgenic plants were similar to each other without stress, while significantly lower EL and MDA contents, together with stronger chlorophyll fluorescence and higher *F*_v_/*F*_m_ ratios, were measured in the transgenic tobacco lines than in the WT after the freezing treatment (Figure 4c–f).

To further investigate the role of *PtrERF9* in cold tolerance, *PtrERF9* was overexpressed in lemon, one of the most cold‐sensitive citrus species; two transgenic lines (#15 and #18) were used for assessing cold tolerance (Figure S3). Both the wild‐type (WT) lemon and transgenic plants grew well under normal growth environment. Upon exposure to freezing at −4 °C for 8 h and a subsequent growth recovery for 5 d at room temperature, the WT plants withered and died, whereas the transgenic plants were still alive despite existence of some wilted leaves (Figure 4g). Consistent with the phenotype, the transgenic plants had lower EL and MDA levels, better chlorophyll fluorescence and higher Fv/Fm ratios in comparison with the WT after the cold stress (Figure 4h–k). All of these results demonstrated that overexpression of *PtrERF9* significantly enhanced cold tolerance in transgenic plants.

### Knockdown of *PtrERF9* in *Poncirus trifoliata* leads to elevated cold sensitivity

**Figure 5:**
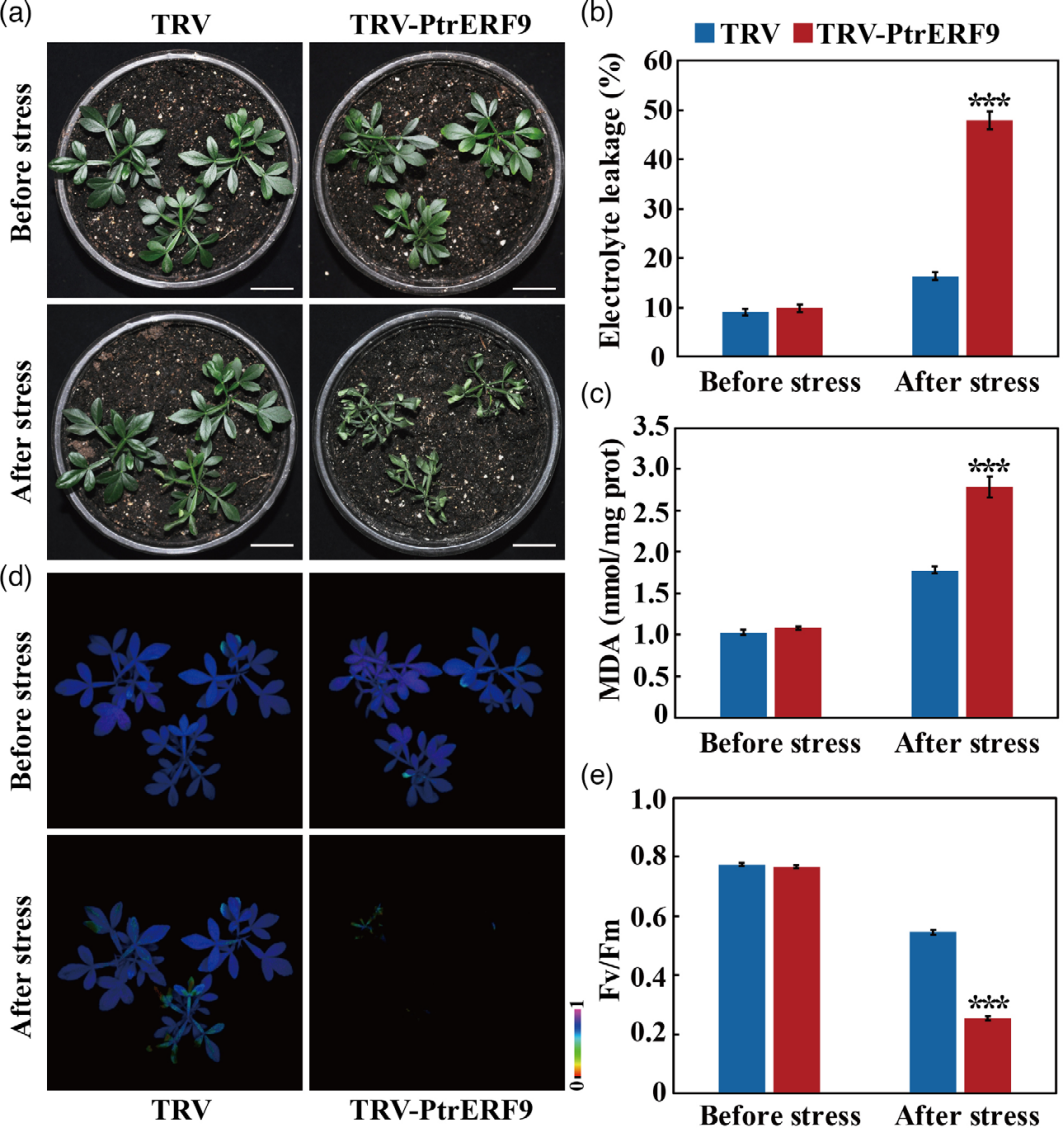
Silencing of *PtrERF9* reduced the cold tolerance in trifoliate orange. (a) Phenotypes of TRV plants and TRV‐*PtrERF9* plants before and after cold treatment. Scale bars = 5 cm. (b–e) Electrolyte leakage (b), MDA content (c), chlorophyll fluorescence (d) and *F*_v_/*F*_m_ (e) of TRV control and TRV‐*PtrERF9* plants before and after the cold treatment. Error bars indicate ± SE (*n* = 3). Asterisks indicate significant difference between the VIGS line and the TRV control plants under same conditions (***P < 0.001).

To further investigate the function of *PtrERF9* in cold tolerance, VIGS was used to knock down *PtrERF9* in *Poncirus trifoliata*. The mRNA abundance of *PtrERF9* in the VIGS plants (defined as TRV‐*PtrERF9*) was sharply reduced compared with the control plants (defined as TRV, Figure S4). Under normal growth condition, there was no difference in plant phenotype between the VIGS plants and the control. After the freezing treatment, the VIGS plants displayed more serious growth inhibition and leaf wilting, whereas the control plants just exhibited slight water‐soaking in the leaves (Figure 5a). In line with the morphology observation, substantially higher EL and MDA content, along with reduced chlorophyll fluorescence and Fv/Fm ratio, were detected in the TRV‐*PtrERF9* plants relative to the control (Figure 5b–e). Moreover, we checked the expression pattern of six other homologous ERF genes in the TRV‐*PtrERF9* plants and found that their mRNA abundance underwent negligible changes when *PtrERF9* was silenced (Figure S5). These results illustrated that VIGS‐mediated knockdown of *PtrERF9* led to enhanced cold sensitivity in trifoliate orange. Taken together, these findings demonstrated that *PtrERF9* plays a positive role in mediating cold tolerance.

### Knockdown of *PtrERF9* causes dramatic alteration of transcriptome

**Figure 6:**
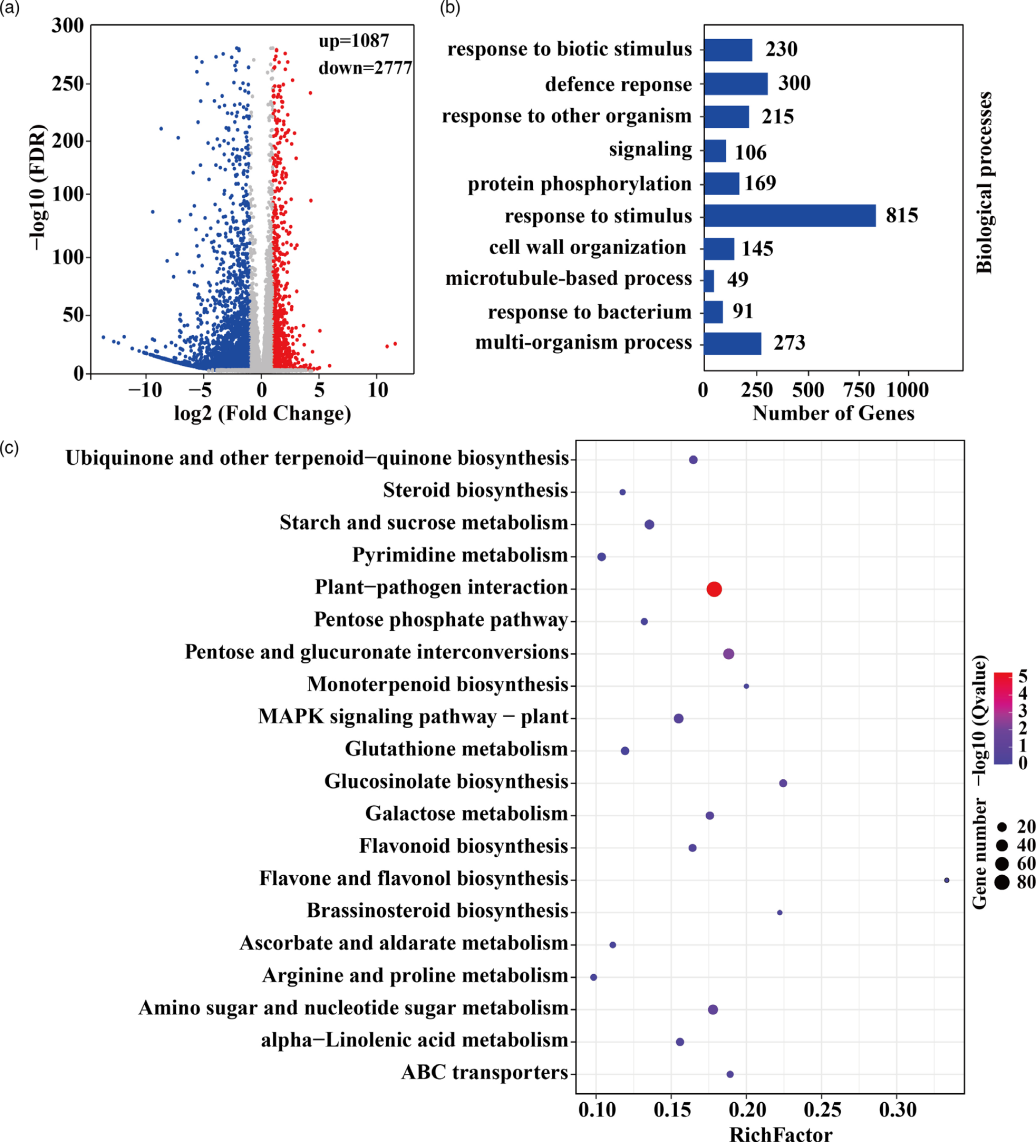
Silencing of *PtrERF9* leads to transcriptional reprogramming of a larger number of genes in trifoliate orange. (a) Scatterplots of gene expression patterns in the VIGS plants compared with TRV control under normal condition. Blue and red circles represent down‐regulated and up‐regulated genes, respectively. (b) GO analysis of enrichment of down‐regulated genes in terms of biological process. (c) The top 20 enriched KEGG pathways among the down‐regulated DEGs.

For further elucidation of the molecular mechanism and identification of downstream target genes of PtrERF9, we performed RNA‐seq using the VIGS and control plants grown under normal conditions. A total of 3864 differentially expressed genes (DEGs), 1087 up‐regulated and 2777 down‐regulated, with significant changes in transcript levels (fold change ≥ 2, *P* ≤ 0.05) were revealed in the VIGS line relative to the control (Figure 6a). To verify the transcriptome profile, expression levels of 12 randomly selected down‐regulated DEGs were analysed by qPCR. The results demonstrated that there is a good correlation between the qPCR and the RNA‐seq data (Figure S6).

We then performed GO and KEGG enrichment analyses to gain deeper insight into the down‐regulated DEGs in the VIGS line. GO analysis showed that the enriched DEGs were involved in various biological processes, with the largest number being grouped into ‘response to stimulus’ (Figure 6b). In addition, 15 out of the top 20 enriched KEGG pathways were related to metabolism or biosynthesis of a range of metabolites, including starch and sucrose, galactose, flavonoid, arginine and proline and glutathione (Figure 6c). In addition, MAPK signalling pathway was also included in the enriched KEGG pathway. An in‐depth analysis of the DEGs showed that some of them are well‐known stress‐related genes, including transcription factors (*ZAT12* and *NAC2*), kinases or phosphatases (*CDPK2* and *PP2C2*), enzymes involved in ROS scavenging (*POD14*, *POD30* and *AO1*) and metabolism (*FAD8*, *UDT76C4*, *GA2ox2* and *PAL)*. Collectively, all these results demonstrated that PtrERF9 possibly regulated a large number of stress‐responsive genes at transcriptional levels to orchestrate the cold stress response.

### *PtrERF9* binds to the promoter of *PtrGSTU17* and activates its expression

**Figure 7:**
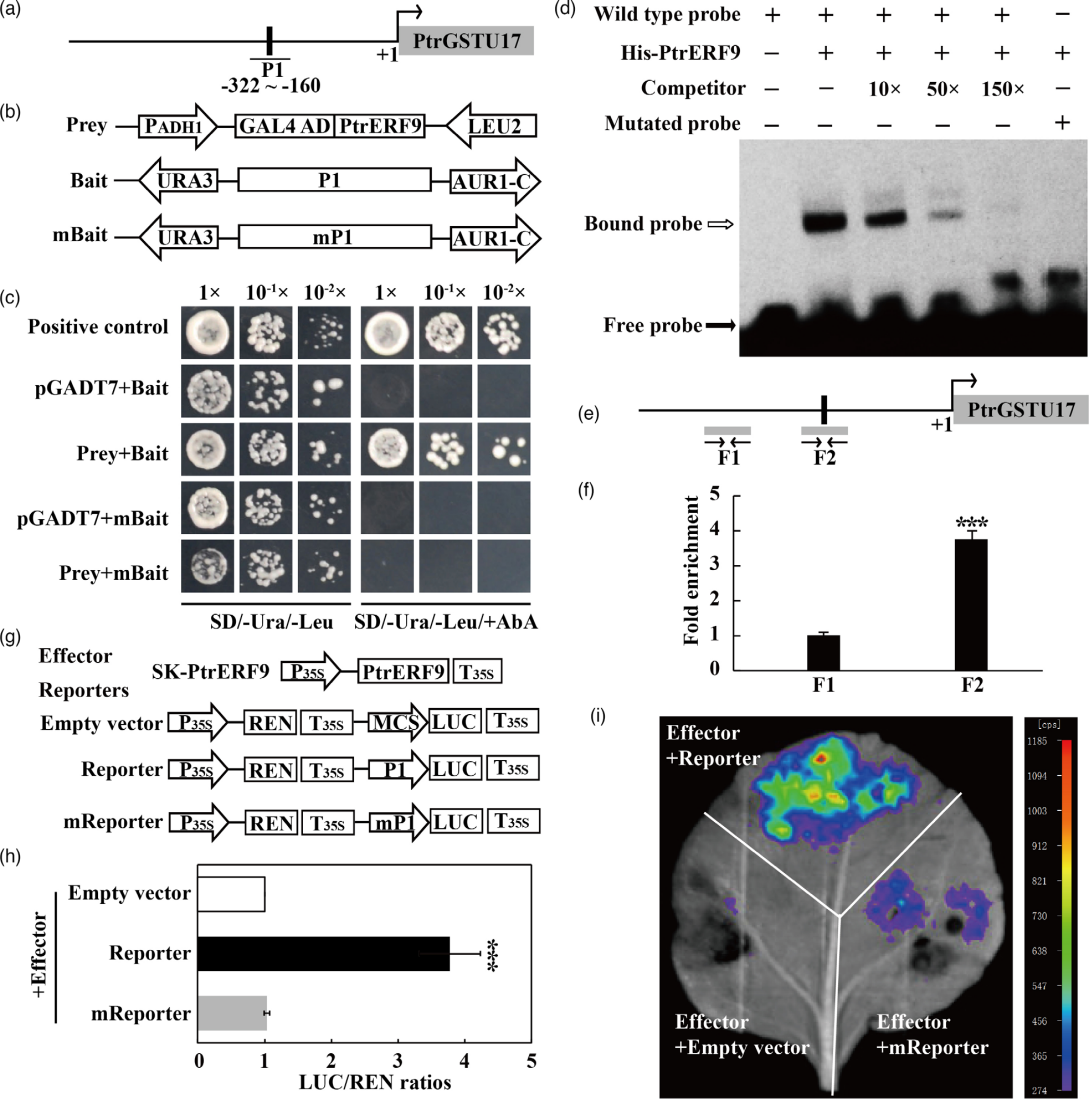
PtrERF9 binds to *PtrGSTU17* promoter and positively regulates its expression. (a) Schematic diagram of the *PtrGSTU17* promoter. The black rectangle indicates the position of the partial promoter fragment (P1) within GCC‐box elements. (b) The prey and bait vectors were used for yeast one‐hybrid assay (Y1H). mP1 is mutated form P1 fragment as the GCCGCC was changed to TCCTCC. (c) Growth of yeast cells co‐transformed with baits (P1 or mP1) and prey combinations (pGADT7‐PtrERF9), positive control (p53‐AbAi + pGAD‐p53), negative control (bait + pGADT7) on SD/‐Ura/‐Leu medium without (left) or with (right) AbA. (d) EMSA assay of specific binding of PtrERF9 to the GCC‐box of the *PtrGSTU17* promoter. The purified His‐PtrERF9 protein was incubated with the biotin‐labelled probe containing the wild‐type or mutated GCC‐box element, with or without unlabelled probe was used as a competitor. Open and closed arrows indicate bound and free probes, respectively. +, presence; −, absence. (e) Schematic diagram of two *PtrGSTU17* promoter fragments (F1, F2), shown as grey bars, used for ChIP‐qPCR analysis. GCC‐box element is marked by black bar. (f) ChIP‐qPCR assays revealed the enrichment of PtrERF9 in the promoter of *PtrGSTU17* by using specific primers as indicated by arrows below the grey bars in (e). (g) Schematic diagrams of the effector and reporter constructs used for dual‐LUC transient expression assay. P_35S_ and T_35S_, the promoter and terminator of CaMV 35S, respectively. MCS, multiple cloning sites. LUC, firefly luciferase. REN, *Renilla* luciferase. (h) Dual‐LUC transient expression assays of the promoter activity were measured by the LUC/REN ratios in tobacco (*Nicotiana benthamiana*) protoplasts. LUC/REN ratio of the negative control (SK‐PtrERF9 + pGreen0800 empty vector) was considered as 1 for normalization. (i) Representative bioluminescence image of the capability of PtrERF9 to regulate the *PtrGSTU17* promoter activity in tobacco leaves. Error bars indicate ± SE (*n* = 3). Asterisks indicate that the value is significantly different from that of the control (***P < 0.001).

Among the DEGs, *PtrGSTU17* (*Pt4g003440*) was found to be tremendously down‐regulated in the VIGS line. In addition, we found that mRNA abundance of *PtrGSTU17* was steadily and progressively increased in the presence of cold treatment, suggesting that *PtrGSTU17* was a cold‐responsive gene (Figure S7). Further analysis demonstrated that the *PtrGSTU17* promoter contained one GCC‐box that is potentially recognized by ERF proteins (Figure 7a). To explore whether PtrERF9 interacts with *PtrGSTU17* promoter, yeast one‐hybrid (Y1H) assay was performed. The yeast cells transformed with the prey and the bait containing P1 fragment harbouring the original GCC‐box, along with the positive control, grew well on the selection medium supplemented with Aureobasidin A (AbA), while mutation of the GCC‐box element (mP1) completely inhibited the yeast growth (Figure 7b,c), indicating that PtrERF9 could interact with the promoter of the *PtrGSTU17* via the GCC‐box core sequence.

In order to further confirm this interaction, we carried out an electrophoretic mobility shift assay (EMSA) using His‐PtrERF9 fusion protein. Incubation of the fusion protein and biotin‐labelled probe containing the wild‐type GCC‐box led to shift of the protein‐DNA complex, which was reduced by adding the unlabelled competitor probe in a dosage‐dependent manner. In addition, no bind shift was detected when the mutated probe was incubated with the fusion protein (Figure 7d). These findings indicate that PtrERF9 directly and specifically bound *in vitro* to the GCC‐box element within the promoter of *PtrGSTU17*.

To examine the *in vivo* binding of PtrERF9 to the *PtrGSTU17* promoter, chromatin immunoprecipitation (ChIP) assay with GFP antibody was performed (Figure S8). The ChIP‐qPCR results revealed that PtrERF9 was significantly enriched in the F2 region of *PtrGSTU17* promoter containing a GCC‐box, but not in the F1 region without the GCC‐box (Figures 7e and f). In addition, we also performed a dual luciferase (LUC) assay to examine whether *PtrERF9* could activate the *PtrGSTU17* promoter. When the effector (SK‐PtrERF9) and the reporter containing the original GCC‐box sequence were transiently co‐expressed in *N. benthamiana* protoplasts, promoter activity, expressed by the LUC/REN ratio, was significantly elevated relative to the control. However, when the GCC‐box was mutated the LUC/REN ratio was resumed to the control level (Figure 7g,h). Quantitative measurement was also supported by microscopic visualization of the LUC fluorescence (Figure 7i). These results demonstrated that PtrERF9 could activate *PtrGSTU17* expression by interacting with the GCC‐box within its promoter.

### Alteration of GST enzymes activity and ROS levels in the transgenic and VIGS plants

**Figure 8:**
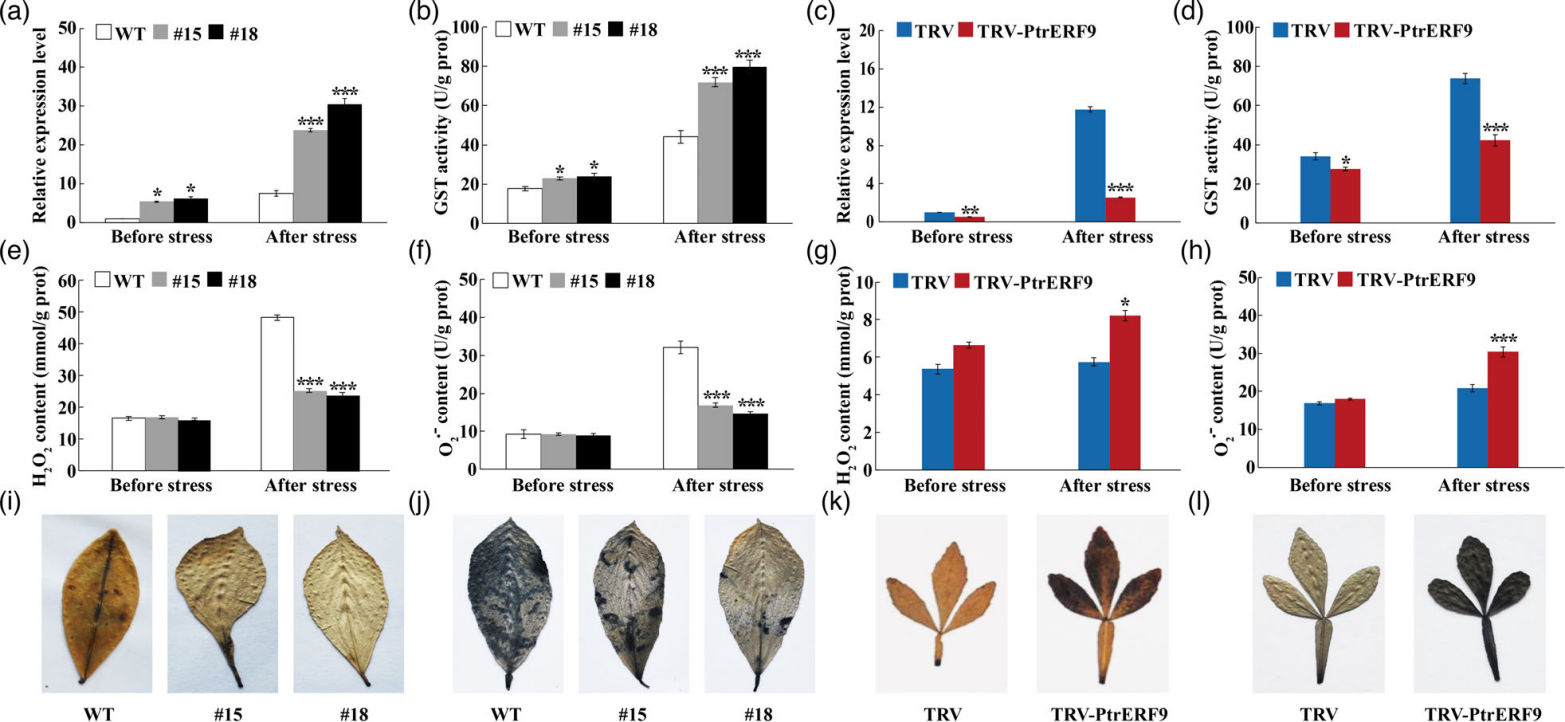
PtrERF9 regulates *PtrGSTU17* expression to control ROS levels in response to cold stress. (a–d) *PtrGSTU17* expression (a, c) and GST activity (b, d) in lemon wild‐type (WT) and transgenic plants (a, b) and TRV control plants and trifoliate orange VIGS (TRV‐PtrERF9) plants (c, d) before and after cold stress. (e–h) Levels of H_2_O_2_ (e, g) and O_2_^·−^ content (f, h) in transgenic lemon and WT (e, f) and TRV control and VIGS plants (g, h) before and after cold treatment. (i–l) *In situ* detection of H_2_O_2_ (i, k) and O_2_^·−^ (j, l) in the WT and transgenic lemons (i, j) and TRV control and VIGS plants (k, l) after cold treatment, as revealed by histochemical staining with DAB and NBT, respectively. Error bars indicate ± SE (*n* = 3). Asterisks indicate significant differences between different groups under the same growth condition (*P < 0.05; ***P < 0.001).

In consideration of interaction and activation of *PtrGSTU17* promoter by PtrERF9, we analysed *GSTU17* expression and GST activity in the plants with overexpression and knockdown of *PtrERF9*. Transcript levels of *GSTU17* and GST enzyme activity were increased in transgenic lemon plants overexpressing *PtrERF9*, but decreased in the VIGS line, relative to the corresponding WT or control in the absence or presence of cold treatment. It is noticed that cold‐induced expression of *PtrGSTU17* and GST activity was more prominent in the *PtrERF9*‐overexpressing lines, but noticeably suppressed in the VIGS plants, when compared to the WT or control (Figures 8a–d).

GST is known to play an essential role in scavenging ROS, including H_2_O_2_ and O_2_^·−^, which are known to be toxic or detrimental to cellular organelles. Therefore, we analysed ROS levels in the tested line. Under normal growth conditions, no profound difference in both H_2_O_2_ and O_2_^·−^ levels was observed between the transgenic or VIGS lines and the WT or control. When subjected to the freezing treatment, H_2_O_2_ and O_2_^·−^ levels were significantly lower in the *PtrERF9*‐overexpressing plants than in the WT. By contrast, the VIGS line contained significantly higher levels of H_2_O_2_ and O_2_^·−^ than the control (Figure 8e–h). Histochemical staining with DAB and NBT is an effective way to detect *in vivo* accumulation of H_2_O_2_ and O_2_^·−^, respectively (Ming *et al*., 2021). The leaves from transgenic plants were stained by DAB and NBT to a much lighter extent, whereas staining of the VIGS line was evidently stronger, when compared to the WT or the control counterparts after the cold treatment (Figures 8i–l). These results showed that accumulation of ROS was dramatically alleviated in the *PtrERF9*‐overexpressing plants, but exacerbated when *PtrERF9* was silenced.

### *PtrERF9* binds to promoter of *PtrACS1* and activates its expression

**Figure 9:**
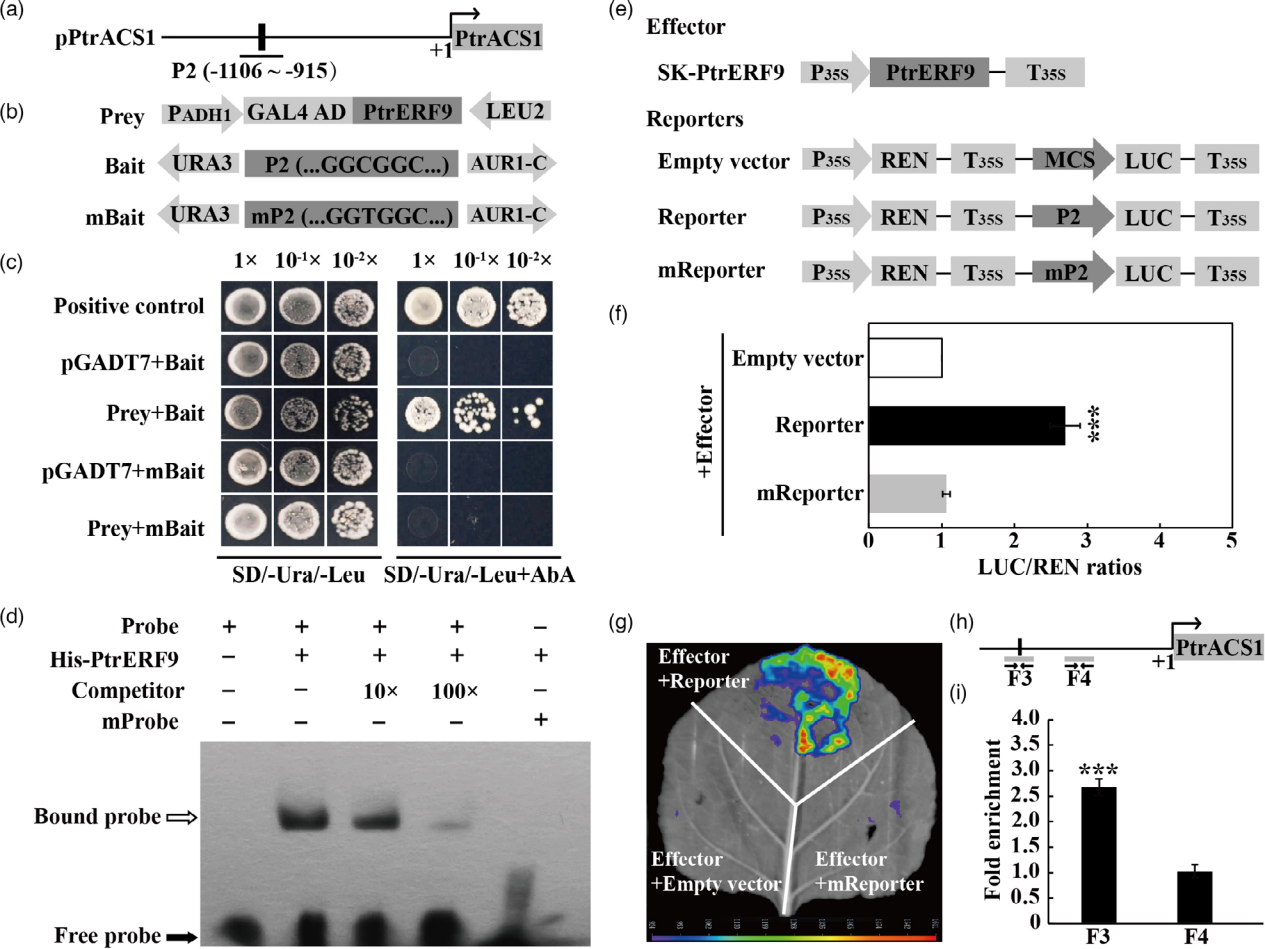
*PtrERF9* binds to *PtrACS1* promoter and positively regulates its expression. (a) Schematic diagram of *PtrACS1* promoter, black rectangle indicates the GCC‐box element in the partial promoter fragment (P2). (b) Prey and bait constructs were used for Y1H analysis, the mutated bait mP2 is a mutated form P2 by changing ‘GGCGGC’ into ‘GGTGGC’. (c) Growth of yeast cells co‐transformed with the prey and bait on selective medium with or without AbA. Positive control: pGADT7‐Rec‐p53 + p53‐AbAi; Negative control: pGADT7 + bait. (d) EMSA assay using purified His‐PtrERF9 fusion protein incubated with biotin‐labelled probe containing wild‐type or mutated probe, along with or without unlabelled competitor DNA. Open and closed arrows indicate bound and free probes, respectively. +, presence; −, absence. (e) Schematic diagrams of effector and reporter constructs used for dual‐LUC transient assay. (f) The various combinations of vectors were performed on Dual‐LUC transient expression assays in tobacco protoplasts. (g) Representative bioluminescence image of PtrERF9 activation on the *PtrACS1* promoter in tobacco leaves. (h) Schematic diagrams of F3 and F4 regions, shown as grey bars, in the *PtrACS1* promoter, in which only F3 contains a GCC‐box sequence (black bar). (i) Enrichment of PtrERF9 in the *PtrACS1* promoter based on ChIP‐qPCR assays using an anti‐GFP antibody. The arrows below the grey bars in (h) show the specific primers used for ChIP‐qPCR. Error bars indicate ± SE (*n* = 3). Asterisks indicate that the value is significantly different from that of the control (***P < 0.001).

Based on the results that ethylene is necessary for cold induction of *PtrERF9*, *PtrERF9* was proposed to be possibly involved in ethylene signalling pathway. Examination of the RNA‐seq database showed that two ACC synthase (ACS)‐encoding genes (*Pt3g038000* and *Pt3g002280*) were substantially repressed in the VIGS line in comparison with the control. However, promoter analysis of the two genes showed that only *Pt3g038000* contained a canonical GCC‐box core sequence within its promoter. *Pt3g03800* and its homologue of *Arabidopsis thaliana* were annotated as the *ACS1* gene in the released genomes of these two plant species, so *Pt3g03800* was named as *PtrACS1* hereafter. In order to illustrate whether *PtrERF9* could regulate *PtrACS1* expression, we investigated interaction between *PtrERF9* and the promoter of *PtrACS1*. Y1H assay showed that the yeast cells co‐transformed with prey and the bait containing the original *PtrACS1* promoter fragment P2 with a genuine GCC‐box sequence grew normally on the selection medium containing AbA, whereas the growth was completely inhibited when the GCC‐box was mutated from GGCGGC to GGTGGC (Figure 9a–c). In addition, EMSA assay showed that an apparent band shift was observed when His‐PtrERF9 fusion protein was incubated with the labelled probe derived from the P2 sequence, whereas inclusion of the competitor DNA in the reaction resulted in a dosage‐dependent suppression of band migration. Furthermore, mutation of the GCC‐box completely abolished the band shift, suggesting that PtrERF9 bound directly and specifically to the GCC‐box within the *PtrACS1* promoter (Figure 9d). Quantitative dual LUC assay and microscopic observation of LUC fluorescence demonstrated that PtrERF9 activated *PtrACS1* though interacting with the GCC‐box, within the promoter, while mutation of the GCC‐box impaired the activation (Figure 9e‐g). In addition, the ChIP‐qPCR assay demonstrated that PtrERF9 was conspicuously enriched in the F3 region of the *PtrACS1* promoter having a GCC‐box sequence, whereas no enrichment was observed in the F4 region without the *cis*‐acting element (Figures 9h,i). All of these results indicate that PtrERF9 regulated *PtrACS1* expression through interacting with the GCC‐box core sequence.

**Figure 10:**
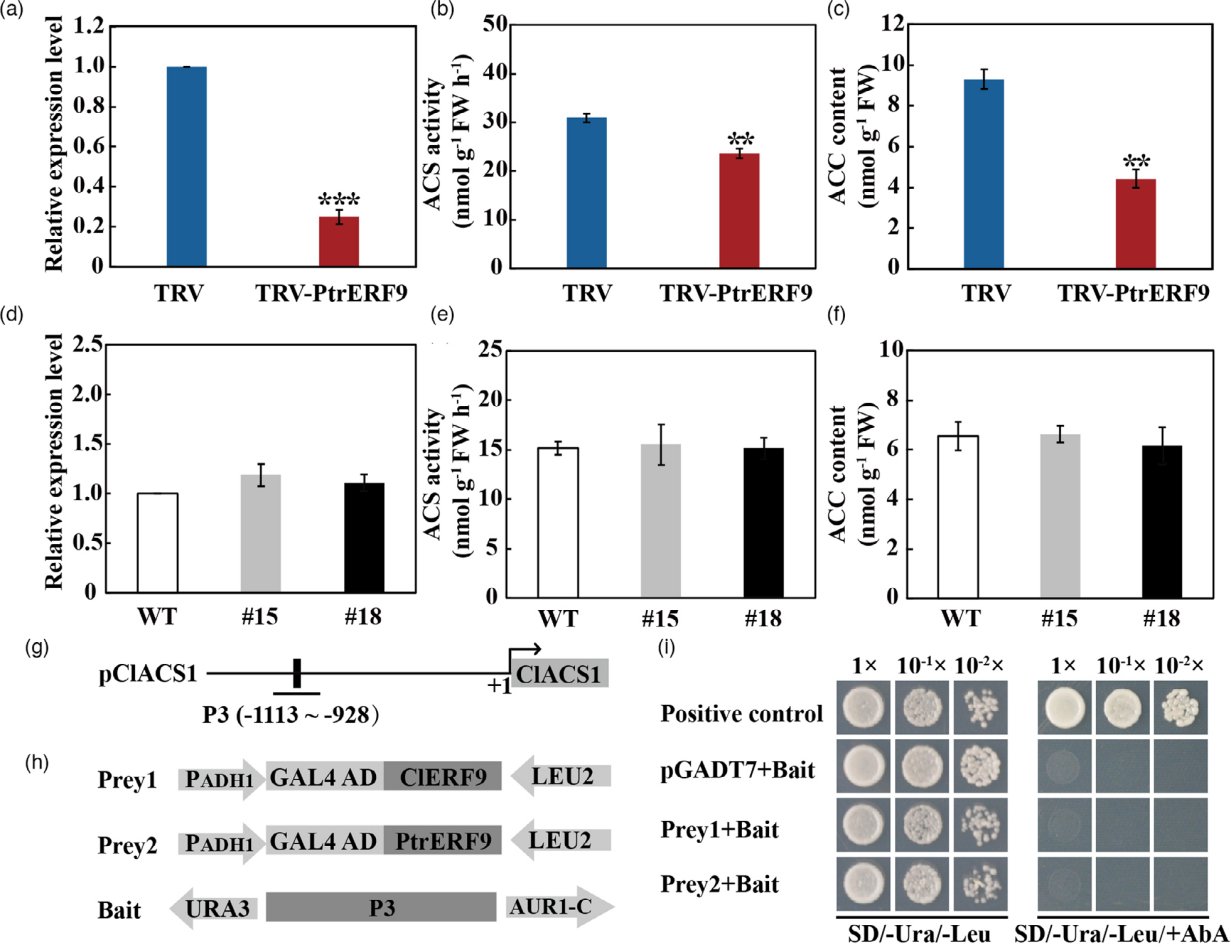
PtrERF9 regulates ethylene synthesis via controlling *ACS* expression in trifoliate orange for cold response. (a–c) *ACS1* expression (a), ACS activity (b) and ACC content (c) in TRV control and *PtrERF9*‐silencing trifoliate orange. (d–f) *ACS1* expression (d), ACS activity (e) and ACC content (f) in wild‐type and *PtrERF9* overexpression transgenic lemon plants. Error bars indicate ± SE (*n* = 3). Asterisks indicate significant differences between different groups under the same growth condition (**P < 0.01; ***P < 0.001). (g) Schematic diagram of *ClACS1* promoter, black rectangle indicated the GGTGGC motif in the partial promoter fragment (P3). (h) Prey and bait constructs were used for yeast one‐hybrid analysis. pGADT7‐PtrERF9 and pGADT7‐ClERF9 were used as prey. P3 fragment was used as bait. (i) Growth of yeast cells co‐transformed with prey (PtrERF9, ClERF9) and bait (P3) on selective medium added with or without AbA. positive control (p53‐AbAi + pGAD‐p53), negative control (bait + pGADT7).

Analysis with qPCR showed that transcript level of *PtrACS1* was drastically down‐regulated in the VIGS line relative to the control (Figure 10a). Consistently, the ACS activity and ACC content were significantly decreased in the VIGS line as compared to the control (Figures 10b,c). Surprisingly, no difference in *ACS1* gene expression, ACS activity and ACC content was detected between transgenic lemon lines overexpressing *PtrERF9* and WT (Figure 10d–f). It seems that PtrERF9 could not regulate *ACS1* gene of lemon (*ClACS1*). In order to confirm this assumption, we first compared the promoter sequences of *ClACS1* and *PtrACS1* as well as the AP2 domains of lemon ERF9 (ClERF9) and PtrERF9. The promoter sequence of *ClACS1* was largely like that of *PtrACS1* except presence of a mutation in the GCC‐box sequence, from GGCGGC in the *PtrACS1* promoter to GGTGGC in the *ClACS1* promoter (Figure S9). By contrast, the sequences of AP2 domains were completely identical between ClERF9 and PtrERF9 (Figure S10). This raised the question of whether the mutation of GCC‐box in the *ClACS1* promoter impeded interaction between ClERF9 or PtrERF9 and *ClACS1* promoter. To answer this question, we carried out Y1H assay to examine interaction between PtrERF9 or ClERF9 and the *ClACS1* promoter. Yeast cells co‐transformed with either ClERF9 or PtrERF9 and the bait constructed using the *ClACS1* promoter fragment P3 containing the GGTGGC sequence could not grow on the selection medium (Figure 10i), implying that *PtrERF9* and *ClERF9* failed to interact with the *ClACS1* promoter. These findings indicate that PtrERF9 and ClERF9 could not regulate the *ClACS1* gene due to mutation of the GCC‐box element within the promoter region.

## Discussion

Plant AP2/ERFs have been known to be involved in plant adaption to various extreme environmental cues, including low temperature, oxidative stress, drought, heavy metals and nutrient starvation (Lin *et al*., 2017; Yao *et al*., 2017; Zhao *et al*., 2020a,b; Zhuo *et al*., 2018). However, the physiological and molecular mechanisms accounting for the functions of most ERFs in cold stress are not well characterized, particularly in perennial plants like *Poncirus trifoliata*. In this study, we characterized an ERF gene, *PtrERF9*, from *P*. *trifoliata*. *PtrERF9* was explored through bioinformatics analysis of the ERF genes based on an earlier RNA‐seq dataset. The cold‐inducible silhouette was further confirmed by analysing expression patterns of the gene and the promoter. In line with the cold‐responsive tribute of *PtrERF9*, we demonstrated that *PtrERF9* functioned as a positive regulator of cold tolerance, as overexpression and knockdown of the gene conferred enhanced and impaired cold tolerance, respectively. Our work adds PtrERF9 as a valuable member for the cold‐responsive gene repository for genetic engineering in an attempt to improve cold tolerance.

The most direct way for transcription factors to exert their functions is the regulation of downstream target genes. In this study, we analysed transcriptome profiles of the VIGS line and the control plants in order to know how knockdown of PtrERF9 influenced gene expression atlas. We predominantly focussed on the down‐regulated genes in the VIGS line, as PtrERF9 plays a positive role in modulation of cold tolerance. It showed that silencing of *PtrERF9* led to alteration of numerous genes (DEGs) involved in a variety of biological processes and molecular functions. Many transcription factors and important components in the signalling network, such as *CDPK* and *PP2C* (Yang *et al*., 2018; Zhu, 2016), were down‐regulated in the VIGS line relative to the control. Of note, GO analysis showed that the biological process ‘response to stimulus’ contained the largest number of DEGs, implying that PtrERF9 acts as a significant regulator of cold stress response. Moreover, KEGG analysis revealed a large number of down‐regulated genes were associated with diverse metabolic pathways, implying that *PtrERF9* may function in cold tolerance by modulating the levels of different metabolites, including fatty acids, flavonoid and phenol, via regulating the relevant genes. In the transcriptome data, we found that *FAD*, *PAL* and *UDPG* genes were substantially down‐regulated in the VIGS line. FAD (fatty acid desaturase) is the key enzyme responsible for biosynthesis of unsaturated fatty acids, which are implicated in modulation of cold tolerance by impacting the membrane fluidity and stabilization (Chen and Thelen, 2013). PAL (phenylalanine ammonia‐lyase) is a rate‐limiting enzyme in the phenylpropanoid pathway associated with the synthesis of secondary metabolites, such as flavonoids and phenols, which have been shown to function in protecting plants from environmental stress (Habibollahi *et al*., 2020). UDPG (UDP‐glycosyltransferase) plays an important role in the biosynthesis of eugenol glucoside or ABA glycosylation, which has been reported to be crucial for coping with abiotic stress (Chen *et al*., 2020; Zhao *et al*., 2020, 2020,2020, 2020). Therefore, PtrERF9 may possibly regulate biosynthesis of these metabolites to impart cold tolerance. However, more extra work is required to clarify this assumption in the future.

It has been documented that plants are well equipped with a highly complex dynamic ROS scavenging system composed of non‐enzymatic and enzymatic antioxidants to combat abiotic stresses, including cold. Thus, ROS homeostasis and relevant antioxidant metabolism are crucial for plant performance and survival under the cold stress. Based on this scenario, we screened the DEGs pertinent to the ROS scavenging and found that expression level of *PtrGSTU17* was tremendously repressed in the VIGS line. GSTU is a plant‐specific GST that has been known to play a critical role in ROS scavenging. Integrative analyses indicated that PtrERF9 directly and specifically bound to the GCC‐box element within the promoter region of *PtrGSTU17* and activated its transcription. This indicates that *PtrGSTU17* is a direct downstream target gene of PtrERF9. To our knowledge, our work is the first to justify transcriptional regulation of a GSTU gene by an ERF protein. Although GST is activated by cold stress, the associated molecular mechanism remains unclear. The illustration of PtrERF9‐PtrGSTU17 module in this study may provide evidence to explain this physiological phenomenon, shedding light on the transcriptional regulation of GST activation in plants exposed to cold stress. In agreement with this regulatory pattern, expression level of *GSTU17* and enzyme activity of GST were increased in the transgenic plants overexpressing PtrERF9, but reduced in the PtrERF9‐silenced line, in comparison with the WT or control plants. Consistent with these alterations, the transgenic plants accumulated significantly less ROS, whereas ROS level was noticeably increased in the VIGS plants, suggesting that ROS scavenging capacity was elevated by overexpression of PtrERF9, but repressed when PtrERF9 was knocked down. Hence, one of the physiological roles of *PtrERF9* in cold tolerance is the modulation of ROS homeostasis through activating *PtrGSTU17* to exert a more robust ROS scavenging capacity.

It is known that ethylene serves as an essential phytohormone involved in cold stress response. Previous studies have demonstrated that ethylene acted as either a positive or a negative regulator of cold tolerance (Shi *et al*., 2012; Sun *et al*., 2016, 2019; Zhang *et al*., 2009; Zhao *et al*., 2014), implying that the role of ethylene in cold tolerance differs among various plant species. In this study, we found cold treatment promoted ethylene production in trifoliate orange. Moreover, exogenous supply of ACC, the precursor of ethylene synthesis, resulted in enhanced cold tolerance, whereas the cold tolerance capacity was greatly impaired by AVG, the inhibitor of ethylene synthesis. These results showed that ethylene plays a positive role in modulation of cold tolerance in trifoliate orange. Numerous studies have demonstrated that ethylene participates in stress response by triggering the well‐defined ethylene signalling pathway (Argueso *et al*., 2007; Tao *et al*., 2015). A few important components are crucial for orchestrating the ethylene‐mediated signalling network, in which the ERFs are important players by relaying the stress signal to downstream stress response. Ethylene‐responsive ERFs involved in cold tolerance have been previously reported in various plants, such as ERF057 of grape (Sun *et al*., 2016), ERF1 of *Medicago falcata* (Zhuo *et al*., 2018) and ERF2 of tomato (Zhang and Huang, 2010). Herein, we also showed that exogenous ACC treatment increased mRNA abundance of *PtrERF9*, and up‐regulation of *PtrERF9* under cold stress was dependent on ethylene. These findings indicated that *PtrERF9* acts downstream of ethylene signalling to regulate its target genes for adaptation to the cold stress.

Ethylene biosynthesis involves two enzymes, ACS and ACC oxidase (ACO), in which ACS has been widely accepted as the rate‐limiting enzyme for ethylene production. In this study, two ACS‐encoding genes, *PtrACS1* and *PtrACS2*, were down‐regulated in the *PtrERF9*‐silencing plants. Further bioinformatics analysis, along with Y1H, EMSA, LUC and ChIP‐qPCR assays, demonstrated that PtrERF9 could directly bind to the GCC‐box element within the promoter of *PtrACS1* and activated its expression. These data indicate that *PtrACS1* served as a direct target gene of PtrERF9. In accordance with this, expression level of *PtrACS1* was dramatically reduced in the *PtrERF9*‐silenced plants, accompanied by lower ACS activity and ACC content, relative to control plants. Earlier studies have proposed that the *ACS* genes were regulated at transcriptional and posttranscriptional levels to control ethylene production under normal and stressful conditions (Catalá *et al*., 2014). Except PtrERF9, several other transcription factors, such as AtWRKY33, AtERF11 of *Arabidopsis thaliana* (Datta *et al*., 2015; Li *et al*., 2011; Zheng *et al*., 2019), ERF1B of apple (Wang *et al*., 2021), have been illustrated as the regulators of *ACS* gene expression and ethylene synthesis in response to various stresses, including pathogen infection, high salinity and cold stress. The facts that ethylene induced expression of *PtrERF9* and that PtrERF9 regulated ethylene synthesis by targeting the *ACS1* gene indicate that PtrERF9 might undergo a positive feedback regulation by ethylene under cold stress. In this regard, *PtrERF9* could be fine‐tuned by ethylene to reinforce its transcriptional regulation of target genes for counteracting the cold stress. Trifoliate orange is extremely cold tolerant, while lemon is one of the most cold‐sensitive species in *Citrus*. Although ClERF9 of lemon had the same AP2 domain sequence as that of PtrERF9, surprisingly, ClERF9 and PtrERF9 could not interact with the promoter of *ClACS1* due to mutation of the GCC‐box sequence. Absence of interaction between ClERF9 and ClACS1 indicates that ethylene synthesis of lemon is possibly weakened under cold stress. As a result, the feedback regulation of ethylene on ClERF9, as observed on PtrERF9, was alleviated. This may provide explanation for the slight induction of ClERF9 under cold stress (Figure S11). Interestingly, the discrepancy in the interaction of ERF9 and ACS1 between trifoliate orange and lemon is largely congruent with cold tolerance capacity of the two species. Therefore, we can assume that cold tolerance may be ascribed, at least partly, to the existence of a regulatory module consisting of ERF9‐ACS1, although other undetermined regulatory modules might also work in this process.

**Figure 11:**
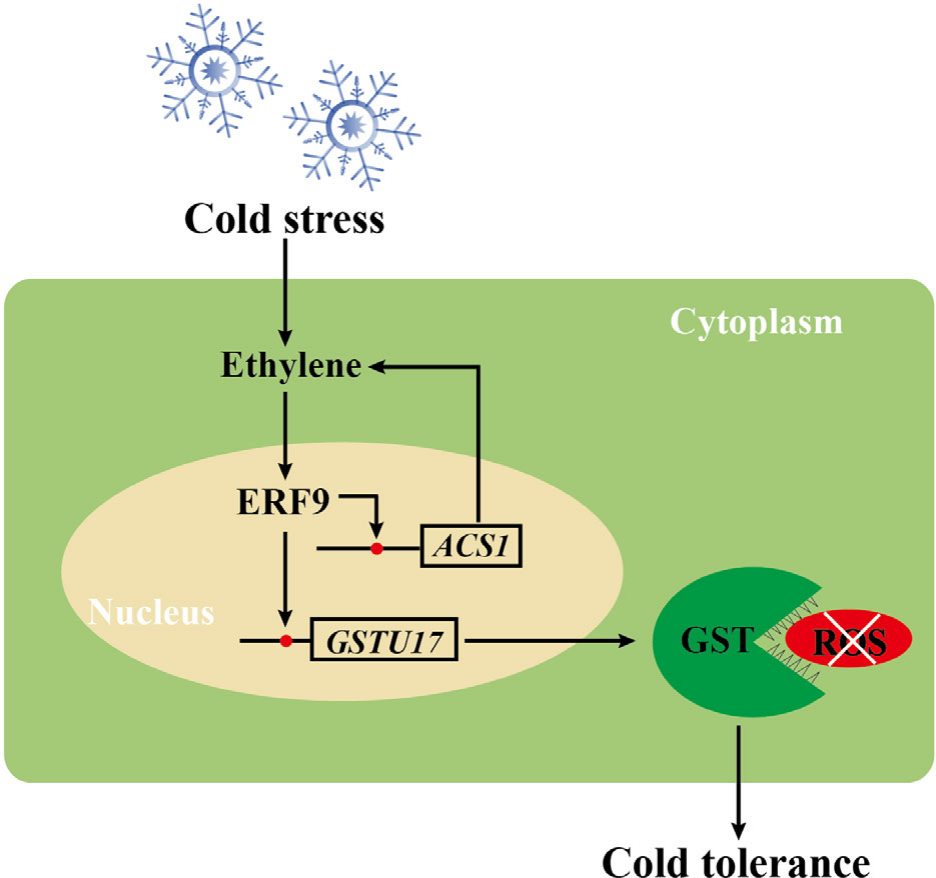
A proposed model for the regulatory function of PtrERF9 under cold stress. Cold stress induces ethylene production and activates the expression of *PtrERF9*. *PtrERF9* binds to the GCC‐box in the promoter of *PtrGSTU17*, improves the plant ROS scavenging captivity. Additionally, *PtrERF9* involves in the signalling pathway and acts as a feedback enhancer to elevate ethylene biosynthesis gene (*PtrACS1*) expression and ethylene level, and finally increases plant cold tolerance. The GCC‐box elements are shown by red circles.

In conclusion, we have demonstrated that PtrERF9 of trifoliate orange, a cold‐responsive ERF transcription factor, plays a positive role in cold tolerance. By using RNA‐seq, we revealed that knockdown of *PtrERF9* led to transcriptome reprogramming of a vast number of genes involved in various biological processes or molecular functions, in which *PtrGSTU17* and *PtrACS1* were confirmed as two downstream target genes of PtrERF9. PtrERF9 functioned in cold tolerance by modulation of ROS homeostasis through regulating PtrGSTU17‐mediated ROS scavenging. In addition, PtrERF9 was responsive to ethylene induction and could regulate ethylene biosynthesis via regulating PtrACS1, forming a feedback regulation loop. Based on our findings, we put forward a working model for *PtrERF9*‐mediated cold tolerance (Figure 11). Cold increases the ethylene production and induces the expression of *PtrERF9*. PtrERF9, on the one hand, binds to the promoter of *PtrGSTU17* and activates its expression to promote ROS scavenging. On the other hand, PtrERF9 induces *PtrACS1* expression and stimulates synthesis of ethylene, which is integrated into the existing ethylene pool to trigger further up‐regulation and activation of PtrERF9 for transcriptionally regulating downstream target genes to promote cold tolerance.

## Materials and methods

### Plant materials and stress treatments

To verify the expression patterns of the *PtrERF9*, we performed qPCR via using the original materials kept at the −80 °C fridge which was used for high‐throughput sequencing as previously described (Wang *et al*., 2015). Wild‐type seeds of trifoliate orange (*Poncirus trifoliata*) and lemon (*Citrus limon*) were obtained from the Citrus Breeding Center at Huazhong Agricultural University. The seeds were grown in soil pots and kept in growth room (16‐h light/8‐h dark cycle) at 25 °C. Two‐month‐old seedlings were used for the following experiments. For ACC treatment, the seedlings were treated with 100 μM ACC, and leaves were collected at 0, 1, 3, 6, 12 and 24 h. For cold treatment, the seedlings were transferred into the growth chamber at 4 °C for 0, 3, 6, 12, 24 and 48 h, and the leaf samples were collected at the designated time points. For the ethylene inhibitor AVG treatment, 100 μM AVG was applied to plants under low‐temperature conditions at 4 °C and the leaves were collected at 0, 3, 6, 12, 24 and 48 h. All samples were immediately frozen in liquid nitrogen and stored at −80 °C until further analyses.

### RNA extraction and qPCR analysis

Total RNA was isolated from the leaves using a TRIzol kit (TaKaRa, Ostu, Shiga, Japan), and the cDNA was synthesized using the RevertAid First Strand cDNA Synthesis Kit (Thermo, Waltham, MA, USA) according to the manufacturer’s instruction. Quantitative real‐time PCR (qPCR) was carried out using a QuantStudio 7 Flex instrument (Applied Biosystems, Foster City, CA, USA) with the SYBR® Green PCR kit (Vazyme, Nanjing, China). PCR reactions were performed in triplicate using 0.2 μM of forward and reverse primers, 5 μL of SYBR qPCR master mix and 200 ng of cDNA in a final volume of 10 μL. Cycling conditions were as follows: 5 min of denaturation at 95 °C, followed by 40 cycles of 95 °C for 15 s and 60 °C for 30 s. *Actin* and *ubiquitin* were used as the normalizing internal controls for citrus and tobacco, respectively. Relative gene expression levels were calculated using the 2^–ΔΔCt^ method, and three replicates were used for all quantitative experiments. The primers for qPCR analysis are listed in Table S1.

### Histochemical assay of GUS activity

A 1431bp promoter fragment upstream of the translation start site (TSS) of *PtrERF9* was amplified with the primers and inserted into DX2181 vector within β‐glucuronidase (GUS) gene to generate pPtrERF9: GUS. The construct was transformed into sweet orange (*Citrus sinensis*) embryogenic callus as described in previous report (Dai *et al*., 2018). After culturing at ambient temperature for 2 d in the dark, the callus was treated at 4 °C for 2 d. Histochemical staining of GUS was performed by the histochemical assay kit (Zhongkeruitai, China) according to the manufacturer's protocol.

### Cloning and sequence analysis of *PtrERF9*

An earlier transcriptome dataset (Wang *et al*., 2015) was analysed to unravel *PtrERFs* involved in cold response. *PtrERF9* (*Pt2g011090*) was selected for further analysis. The full‐length coding sequence of *PtrERF9* was amplified from *Poncirus trifoliata* leaf cDNA with the primers *Pt2g011090*‐F/R (Table S1) by PCR. The sequence analysis was performed to construct phylogenetic tree and multiple alignments by using MEGA X and GENEDOC software, respectively.

### Subcellular localization analysis

The *PtrERF9* full‐length CDS without the stop codon was amplified with primers containing *Eco*RI and *Bam*HI restriction sites and then fused to the 101LYFP vector, under the control of the CaMV 35S promoter. The construct (35S: PtrERF9‐YFP) or the control vector (35S: YFP) was co‐transformed with the nuclear marker VirD2NLS‐mCherry into *N. benthamiana* leaves by *Agrobacterium tumefaciens*‐mediated transformation as described previously (Sparkes *et al*., 2006). DAPI was also used for staining of nucleus. The infiltrated plants were kept in growth chamber for another two days, and the fluorescent signals were observed with a laser scanning confocal microscope (Leica TCS‐SP8, Wetzlar, Germany).

### Vector construction and plant transformation

For the *PtrERF9* overexpression lines, the full‐length *PtrERF9* CDS sequence was cloned into the pGWB411 vector according to the gateway recombination technology (Invitrogen). The resulting construct was transformed into tobacco (*Nicotiana nudicaulis*) and lemon (*C. limon*) via *Agrobacterium*. *tumefaciens*‐mediated transformation as previously reported (Fu *et al*., 2011). And then the transgenic explants were grown on MS (for tobacco; Murashige and Skoog, 1962) or MT (for lemon; Murashige and Tucker, 1969) medium containing 50 μg/mL kanamycin. Kanamycin‐resistant plants were selected which were further confirmed by qPCR assay. The transgenic tobacco lines at T_2_ generation and lemon lines in the vegetative period were used for phenotype analysis. Similarly, transient expression of *PtrERF9* in trifoliate orange was performed according to Acanda *et al*. (2021) with minor modifications. To this end, full‐length cDNA of *PtrERF9* was cloned into the pH7WG2D expression vector with a GFP tag. The *35S*: *PtrERF9*‐GFP construct was transformed into *Agrobacterium* strain (EHA105) and then used for injection into the young tender leaves of three‐month‐old trifoliate orange seedlings. After 12 days of agroinfiltration, the infected leaves were subjected to fluorescence observation using a hand‐held lamp providing dual‐wavelength fluorescent protein excitation light source (LUYOR‐3415RG, USA). The leaves emitting the green fluorescence were used for Western blotting and ChIP‐qPCR assays.

### Virus‐induced gene silencing

The VIGS system with the tobacco rattle virus was used to decrease *PtrERF9* expression in trifoliate orange plants. A 390‐bp fragment of the *PtrERF9* was amplified and inserted into pTRV2 vector to generate the pTRV2‐*PtrERF9* construct. The vectors pTRV1, pTRV2 and pTRV2‐*PtrERF9* were transformed into *Agrobacterium tumefaciens* strain GV3101. The *Agrobacterium* mixture containing pTRV1, pTRV2 or pTRV2‐*PtrERF9* (v: v, 1: 1) was infiltrated into the germinating seeds of trifoliate orange with shoots (around 2 cm) as described previously (Dai *et al*., 2018; Wang *et al*., 2019). After infiltration, the plants were maintained at 25 °C in a growth room for 3 days under darkness and then the plants were washed with distilled water and transplanted into soil. After four weeks, the leaves of seedlings were subjected to qPCR assay to select the silenced plants for further analyses.

### Cold tolerance assays

For cold stress, one‐month‐old transgenic tobacco and wild‐type (WT) seedlings were directly exposed at −4 °C for 6 h, followed by recovery at 25 °C for 3 d. In addition, for lemon, the wild‐type plans and transgenic lines were treated for 8 h at −4 °C, and then recovered for 5 d at ambient temperature. As for the VIGS trifoliate orange plants, two‐month‐old TRV control plants and the TRV‐*PtrERF9*‐VIGS lines were subjected to −4 °C treatment for 12 h and then recovered for 3 d at ambient temperature. In addition, two‐month‐old health wild‐type seedlings of trifoliate orange were transferred into triangle flasks containing water, or 100 μM ACC, or 100 μM AVG solution for 2 h prior to exposure at −5 °C for 9 h, followed by growth recovery for 2 d at 25 °C. The leaves of tested plants were harvested before or after cold treatment and then used for physiological analysis.

### Physiological measurement and histochemical staining

Electrolyte leakage was measured as described in previous report (Dahro *et al*., 2016). MDA content, H_2_O_2_ content, O_2_^·−^ level and GST activity were measured by using the relevant detection kits (A003‐1 for MDA; A064 for H_2_O_2_; A052 for O_2_^·−^, A004‐1 for GST activity, Nanjing Jiancheng Bioengineering Institute, China) according to manufacturer’s instructions. Total protein levels were tested using the Coomassie Brilliant Blue G‐250 staining method as previously described (Bradford, 1976). H_2_O_2_ and O_2_.^−^ were also checked by histochemical staining with DAB and NBT as previously reported (Huang *et al*., 2013). The chlorophyll fluorescence was detected by using an IMAGING‐PAM chlorophyll fluorometer (Walz, Germany), and the Imaging WinGege software was used to measure the *F*_v_/*F*_m_ ratio.

### RNA‐seq analysis

High‐throughput RNA‐sequencing was used to identify downstream target genes of *PtrERF9*. Total RNA was extracted by TRIzol kit (TaKaRa, Japan) from leaves of *PtrERF9*‐silenced and TRV control plants according to the user manual. Three biological replicates were used for each genotype sample, and the individual total RNA quality and concentration were checked by Agilent 2100 Bioanalyzer and NanoDrop 2000 (Thermo Scientific). The cDNA libraries were constructed and then sequenced with BGISEQ‐500 platform, which was processed by the BGI Company (Shenzhen, China). The raw reads were filtered to remove reads with > 5% unknown nucleotides and low‐quality reads (*Q*‐value < 10). Then, the clean reads were mapped to the *Poncirus trifoliata* genome (Huang *et al*., 2021) by using HISAT and Bowtie2 software. The gene expression levels were calculated by FPKM method and the NOISeq software was used to identify differentially expressed genes in pair‐wise comparisons as described by Tarazona (Tarazona *et al*., 2015). Genes with expression fold change ≥ 2 and false discovery rate (FDR) < 0.05 were defined as DEGs. Gene ontology categories represented by DEGs were performed by the GOseq analysis (Young *et al*., 2010), and Kyoto Encyclopedia of Genes and Genomes (KEGG) classifications were performed using KABAS software as previously described (Mao *et al*., 2005).

### Yeast one‐hybrid assay

Yeast one‐hybrid assay was used to check the binding of *PtrERF9* to the *PtrGSTU17*/*PtrACS*1/*ClACS1* promoter and was also used for testing the interaction between *ClERF9* with the *ClACS1* promoter. The *PtrERF9/ClERF9* coding sequence was cloned into the *Nde*I‐*Eco*RI site of the pGADT7 vector as preys. The promoter fragment within GCC‐box (P1 for *PtrGSTU17*, −160 to −322, P2 for *PtrACS1*, −915 to −1106, P3 for *ClACS1*, −928 to −1113) and mP1 and mP2 mutated forms of P1 (GCCGCC mutated to TCCTCC) and P2 (GGCGGC mutated GGTGGC) was amplified and inserted into the pAbAi vector as baits. These preys and baits were combined and co‐transformed into yeast Y1H Gold strain using the Matchmaker Y1H library screening system (Clontech, Mountain View, CA, USA) following by the manufacturer’s protocol. Positive control (pGAD‐p53 + p53‐AbAi) and negative control (pGADT7‐AD + bait) control were also handled in the same method.

### Electrophoretic mobility shift assay

The full‐length *PtrERF9* was cloned into the expression vector pHMGWA; then, the construct was transformed into *Escherichia coli* strain Rosetta (DE3). The fused protein (His‐PtrERF9) was induced by 0.05 mM isopropyl‐β‐D‐1‐thiogalactopyranoside and purified using Ni‐NTA Agarose according to the manufacturer’s protocol (Qiagen, Germany). The probes containing GCC‐box or mutated elements were synthesized and labelled with biotin by Tsingke Biological Technology (Beijing, China). Unlabelled probe was used as the competitor. EMSA was performed using the LightShift Chemiluminescent EMSA Kit (Pierce). All DNA‐binding reactions were carried out in 1× binding buffer containing 5 mM MgCl_2_, 50 mM KCl, 10 mM EDTA, 2.5% glycerol, 50 ng/μL Poly (dI‐dC) and 0.05% NP‐40. The reaction mixtures were separated on 6.5% nondenaturing polyacrylamide gel; then, gel was electroblotted onto the nylon membrane (Biosharp, Hefei, China), followed by chemiluminescence detection.

### Chromatin immunoprecipitation‐qPCR assay

Immunoblot assays were performed as described by Acanda *et al*. (2021). The protein was incubated with anti‐GFP antibody (Abbkine, a02020, Wuhan, China) or actin antibody (ABclonal, ac009, Wuhan, China) and was then detected with HRP goat anti‐mouse IgG (H + L) antibody (ABclonal, aS003). ChIP‐qPCR assay was performed following the procedure as described previously (Ming et al., 2021). In brief, approximately 1 g of leaves collected from the PtrERF9‐GFP‐overexpressing or wild‐type plants was cross‐linked in 1% (w/v) formaldehyde, and the reaction was stopped by adding 125 mM glycine. After sonication, the anti‐GFP mAb‐magnetic agarose beads (MBL, Nagoya, Japan) were used to immunoprecipitate the chromatin. The recovered DNA was used to performed qPCR by using specific primers (Table S1). The fold enrichment of DNA fragments was calculated according to Chen *et al*. (2018).

### Dual luciferase assay

The CDS of *PtrERF9* was inserted into pGreen 62‐SK vector to generate the effector, and the original and mutated promoter fragments of *PtrGSTU17* or *PtrACS1* were recombined to the pGreen 0800‐LUC vector to generate reporters (Hellens *et al*., 2005). The recombinant plasmids were separately transformed into *N. benthamiana* leaves via the *A*. *tumefaciens*‐mediated transformation. The isolation of tobacco protoplasts was used to analyse transient expression. Luciferase activities were measured with Dual‐Luciferase® Reporter Assay Kit (Promega, Madison, WI, USA) according to the manufacturer’s instruction. Fluorescence was measured using an Infinite 200 Pro microplate reader (Tecan, Mannedorf, Switzerland) and NightSHADE LB 985 (Berthold, Germany).

### Measurement of ethylene production

Ethylene was measured following previous description (Sun *et al*., 2016) with some minor modifications. Two‐month‐old trifoliate orange seedlings were incubated in beakers containing sterile deionized water under normal growth condition (16‐h light/8‐h dark cycle, 25 °C) for 3 d. Then, the plants were transferred to a growth chamber set at 4 °C. The seedlings were taken out at 0, 3, 6, 12, 24 and 48 h for leaves collection. Each plant leaves with petioles were weighed and put into 20 mL sealed bottle containing wet cottons. After 24 h room temperature incubation, 1 mL of gas was withdrawn with a syringe from each bottle and injected into a gas chromatograph (Agilent 7890B, Palo Alto, CA, USA) to quantify the ethylene production. ET production (μg/g/h) was determined in comparison with a standard curve of ethylene. All measurements were gained in triplicate from three independent samples.

### Measurement of ACC content

Determination of ACC content was performed according to protocols described previously (Li *et al*., 2020) with some modifications. The leaf of plants was ground with liquid nitrogen, and about 0.05 g of tissues powder was placed into 2 mL tube and then vortexed with 1.5 mL 80% ethanol. The mixture was incubated at 80 °C for 15 min, and then was centrifuged for 15 min at 8000 ***g***. The supernatant was transferred to a new 2 mL centrifuge tube with 0.5 mL 80% ethanol and then vortexed for 1 min. The content was incubated at 80 °C for 15 min and then centrifuged at 8000 ***g*** for 15 min. The supernatant was merged and dried in a Speed Vac (Labconco, Kansas City, MO, USA), and then 2 mL double‐distilled H_2_O with 0.5 mL CHCl_3_ was added to the tube to resuspend the dried product for 5 min. Finally, the mixture was centrifuged at 4000 ***g*** for 15 min to extract ACC in aqueous phase. Transfer 0.8 mL of suspended in two separate 20 mL vials (one of them to be used for the reaction, the other was added 20 μL 50 μM ACC as an internal standard), then 0.4 mL 50 mM HgCl_2_ was added into both vials. The vials were sealed with a serum cap. Then, 0.2 mL of 5% NaClO and saturated NaOH (2 : 1, v/v) were injected into the vials, which were vortexed for 10 s and incubated on ice for 15 min. One mL of the head space gas was sampled and assayed for ethylene as described above. The ACC content was calculated from the reaction efficiency of ACC converted into ethylene.

### Measurement of ACS activity

ACS activity was measured according to previous protocol (Zhao *et al*., 2013) with minor modifications. Plants were frozen in liquid nitrogen, and the 0.05 g tissue was ground with 2 mL 0.1 M KBS buffer (pH = 8) and then was gently shaken 30 min at 4 °C. The homogenate was centrifuged for 30 min at 4000 ***g*** at 4 °C, and the supernatant was collected for ACS activity measurement. The 0.5 mL extract was mixed with 1.5 mL reaction buffer containing 0.05 M KBS (pH = 8), 250 μM SAM, 1 mM PLP; then, the mixture was placed into a 20 mL bottle with a rubber stopper and incubated 1 h at 30 °C. The purified extract was reacted with chlorinated SAM, and the amount of ACC formed was quantified by chemically converting it to ethylene. The following step of ACC measurement was performed according to the method described above.

### Statistical analysis

All the experiments were repeated at least three times for each study. Statistical differences were evaluated using one‐way analysis of variance (ANOVA) method in SPSS (IBM, NY) based on a *t* test, taking * *P* < 0.05, ** *P* < 0.01 and *** *P* < 0.001 as significant.

## Conflict of interest

The authors declare no conflict of interest.

## Author contributions

J.H.L. and Y.Z. conceived and designed the research. Y.Z. performed the experiments. Y.W. assisted bioinformatics analysis. Y.Z., R.M., M.K., B.D. and W.X. analysed the data. Y.Z. and C.L. wrote the manuscript draft, J.H.L. finalized writing and revision of the manuscript. All authors have read and approved the final version of the manuscript.

## Supporting information


**Figure S1** Phylogenetic analysis and sequence alignments of PtrERF9 and ERFs from *Poncirus trifoliata* and other plants.
**Figure S2** Generation and molecular identification of transgenic tobacco plants overexpressing *PtrERF9*.
**Figure S3** Generation and molecular Identification of transgenic lemon plants overexpressing *PtrERF9*.
**Figure S4** Molecular characterization of the *PtrERF9*‐VIGS plants.
**Figure S5** Expression levels of six ERF genes in TRV control and *PtrERF9*‐silencing trifoliate orange.
**Figure S6** Validation of differentially expressed genes by qPCR analysis.
**Figure S7** Relative expression of *PtrGSTU17* at the designated time points of cold treatment by qPCR.
**Figure S8** Analysis of PtrERF9‐GFP protein level in the *Poncirus trifoliata* leaves transiently expressing *PtrERF9* by western blot.
**Figure S9** Comparison and analysis of *PtrACS1* and *ClACS1* promoters.
**Figure S10** Sequence alignments of PtrERF9 and ClERF9.
**Figure S11** Expression levels of *ClERF9* from *Citrus limon* under cold treatment.
**Table S1** List of primers used in this study.
**Table S2** Summary of RNA‐seq results.

**Table S3** Up‐ and down‐regulated DEGs in RNA‐seq.
