# Supplementary material for: ERF9 of Poncirus trifoliata (L.) Raf. undergoes feedback regulation by ethylene and modulates cold tolerance via regulating a glutathione S‐transferase U17 gene
Source: Plant Biotechnol J. 2021 Sep 29;20(1):183–200. doi: 10.1111/pbi.13705 (PMC8710834; doi:10.1111/pbi.13705)
Supplement: Supplementary file 1 — Figure S1 Phylogenetic analysis and sequence alignments of PtrERF9 and ERFs from Poncirus trifoliata and other plants. Figure S2 Generation and molecular identification of transgenic tobacco plants overexpressing PtrERF9. Figure S3 Generation and molecular Identification of transgenic lemon plants overexpressing PtrERF9. Figure S4 Molecular characterization of the PtrERF9‐VIGS plants. Figure S5 Expression levels of six ERF genes in TRV control and PtrERF9‐silencing trifoliate orange. Figure S6 Validation of differentially expressed genes by qPCR analysis. Figure S7 Relative expression of PtrGSTU17 at the designated time points of cold treatment by qPCR. Figure S8 Analysis of PtrERF9‐GFP protein level in the Poncirus trifoliata leaves transiently expressing PtrERF9 by western blot. Figure S9 Comparison and analysis of PtrACS1 and ClACS1 promoters. Figure S10 Sequence alignments of PtrERF9 and ClERF9. Figure S11 Expression levels of ClERF9 from Citrus limon under cold treatment. Table S1 List of primers used in this study. Table S2 Summary of RNA‐seq results. [file PBI-20-183-s001.docx]

**ERF9 of *Poncirus trifoliata* (L.) Raf. undergoes feedback regulation by ethylene and modulates cold tolerance through regulating a *glutathione S-transferase U17* gene**

**Yang Zhang, Ruhong Ming, Madiha Khan, Yue Wang, Bachar Dahro, Wei Xiao, Chunlong Li*, Ji-Hong Liu***

Supporting Information

The following Supporting Information is available for this article:

**Figure S1.** Phylogenetic analysis and sequence alignments of PtrERF9 and ERFs from *Poncirus trifoliata* and other plants.

**Figure S2.** Generation and molecular identification of transgenic tobacco plants overexpressing *PtrERF9*.

**Figure S3.** Generation and molecular identification of transgenic lemon plants overexpressing *PtrERF9*.

**Figure S4.** Molecular characterization of the *PtrERF9*-VIGS plants.

**Figure S5.** Expression level of six *ERF* genes in TRV control and *PtrERF9*-silencing trifoliate orange.

**Figure S6.** Validation of differentially expressed genes by qPCR analysis.

**Figure S7.** Relative expression of *PtrGSTU17* at the designated time points of cold treatment by qPCR.

**Figure S8.** Analysis of PtrERF9-GFP protein level in the *Poncirus trifoliata* leaves transiently expressing *PtrERF9* by western blot.

**Figure S9.** Comparison and analysis of *PtrACS1* and *ClACS1* promoters.

**Figure S10.** Sequence alignments of PtrERF9 and ClERF9.

**Figure S11.** Expression levels of *ClERF9* from *Citrus limon* under cold treatment.

**Table S1.** List of primers used in this study.

**Table S2.** Summary of RNA-seq results.

**Table S3.** Up- and down-regulated DEGs in RNA-seq.


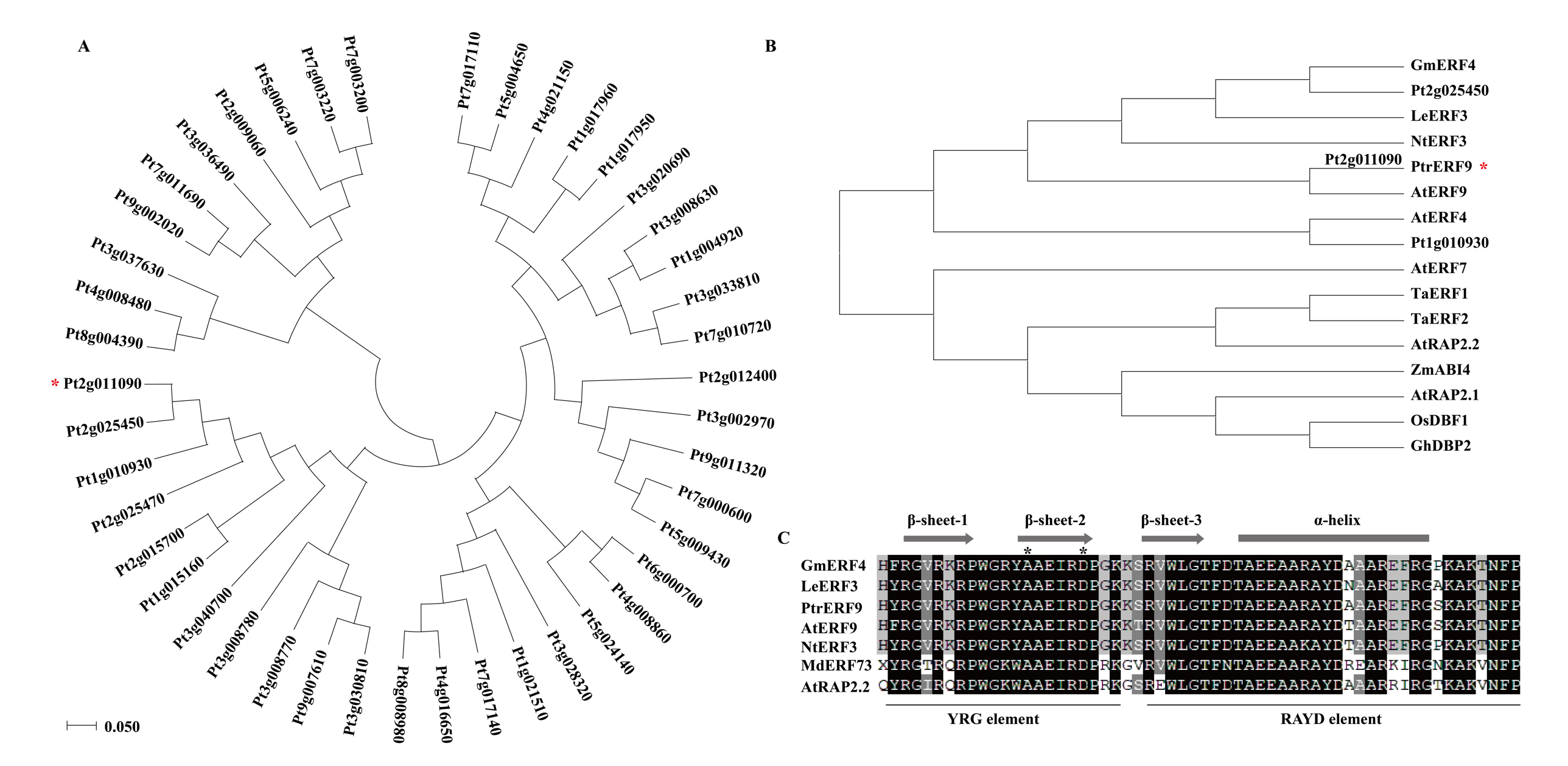


**Figure S1.** **Phylogenetic analysis and sequence alignments of PtrERF9 and ERFs from *Poncirus trifoliata* and other plants.** (A) The phylogenetic tree with 44 ERFs from *Poncirus trifoliata*. The Pt2g011090 protein was marked with a red asterisk. (B) The phylogenetic tree of PtrERF9 protein (marked with a red asterisk) and ERFs from other plant species. (C) Multiple alignments of the AP2 domains of PtrERF9 and ERFs from other plants. Identical and conserved amino acid residues are shown in black and gray shade, respectively. The alanine and aspartic acid residues at positions 14 and 19 are marked by black asterisks. The grey arrows and bar indicate β-sheets and α-helix regions, respectively. The plant species and Gene ID were as follows: *Arabidopsis thaliana* AtERF4 (AT3G15210), AtERF7 (AT3G20310), AtERF9 (AT5G44210), AtRAP2.1 (AT1G46768) and AtRAP2.2 (AT3G14230); *Lycopersicon esculentum* LeERF3 (AY192369); *Nicotiana tabacum* NtERF3 (AB573716); *Glycine max* GmERF4 (ACE76905); *Gossypium hirsutum* GhDBP2 (AY619718); *Triticum aestivum* TaERF1 ([AY271984](https://www.ebi.ac.uk/ena/browser/view/AY271984)) and TaERF2 ([A0A3B6TFN7](https://www.uniprot.org/uniprot/A0A3B6TFN7)); *Zea mays* ZmABI4 (AY125490); *Oryza sativa* OsDBF1 (AP004727); and *Malus domestica* MdERF73 (XP_008369034).


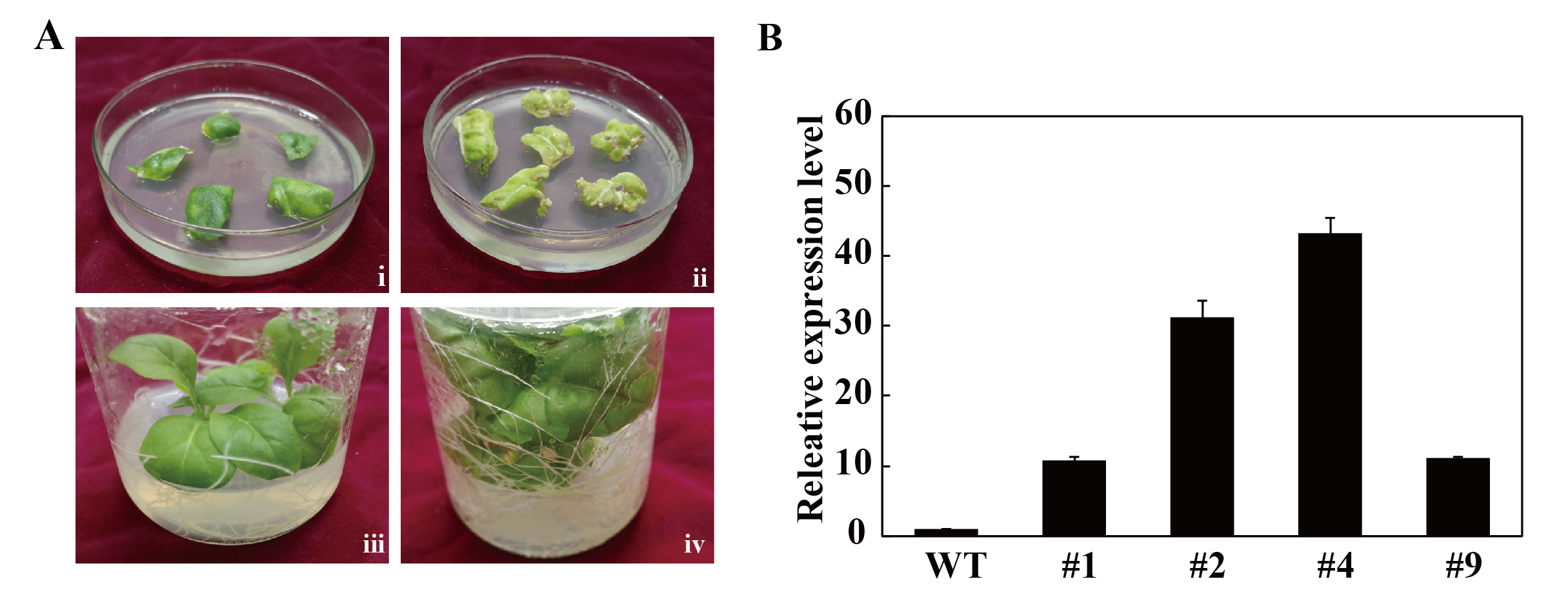


**Figure S2. Generation and molecular identification of transgenic tobacco plants overexpressing *PtrERF9*.** (A) Transformation and regeneration of transgenic tobacco. (A)-ⅰ Tobacco leaf pieces were grown on co-culture medium. (A)-ii Growth of shoots on the selection medium with kanamycin; (A)-iii Generation and proliferation of kanamycin-resistant shoots. (A)-iv Rooting plants. (B) Expression analysis of *PtrERF9* in transgenic tobacco by qPCR. Ubiquitin was used as an internal control. Error bars indicate ± SE (n = 3).


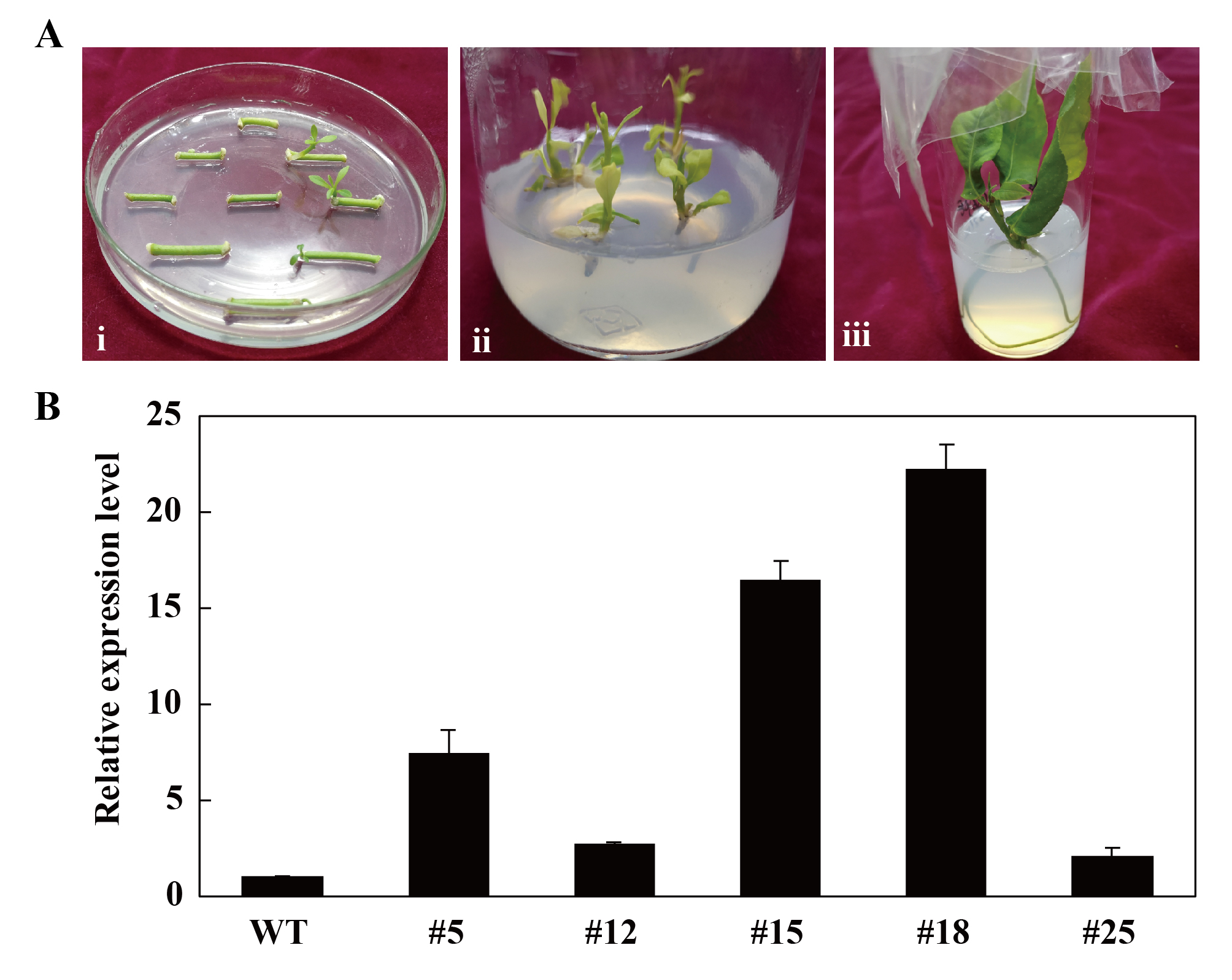


**Figure S3. Genetic transformation and molecular identification of *PtrERF9* overexpression lemon.** (A) The transformation and regeneration of lemon: (A)-i Shoot segments on the shooting medium; (A)-ii Elongation and multiplication of shoots; (A)-iii Rooted plants. (B) qPCR was used to analyze the *PtrERF9* expression of transgenic lemon, using Actin as an internal control. Error bars indicate ± SE (n = 3).


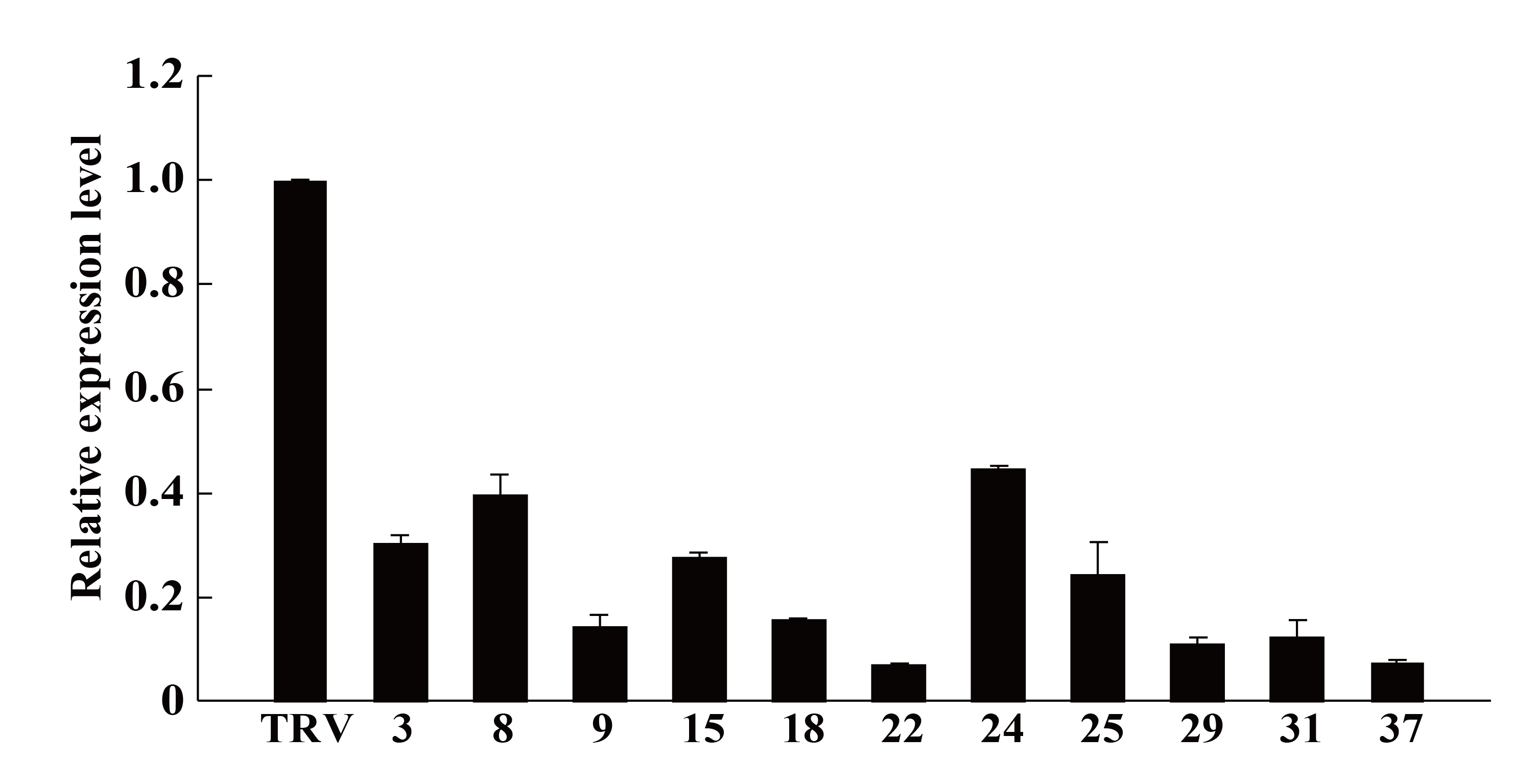


**Figure S4. Molecular identification of VIGS plants.** qPCR was used to analyze the *PtrERF9* expression of TRV2-*PtrERF9* plants, using. *Actin* was used as an internal control. Error bars indicate ± SE (n = 3).


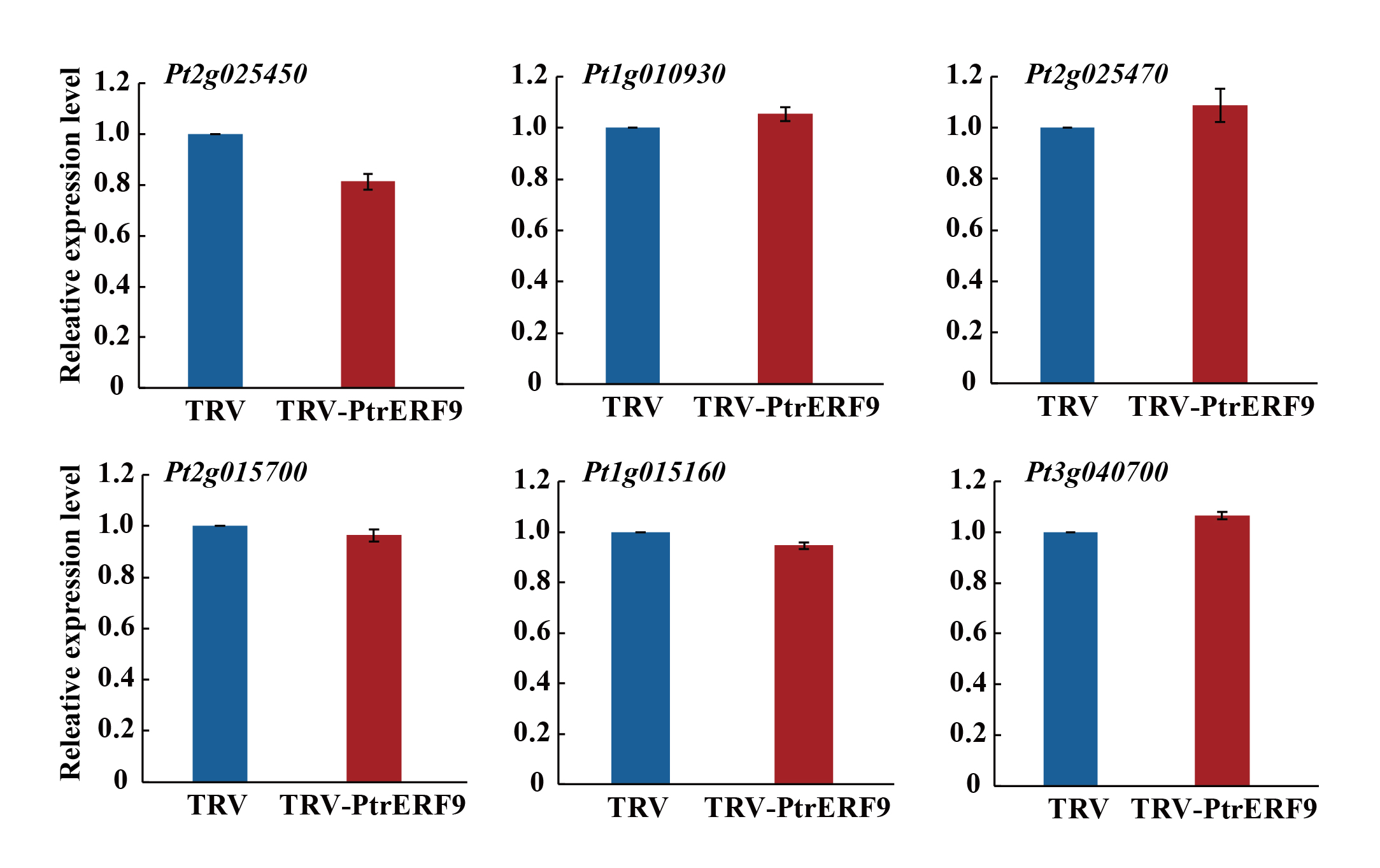


**Figure S5. Expression levels of six ERF genes in TRV control and *PtrERF9*-silencing trifoliate orange.**


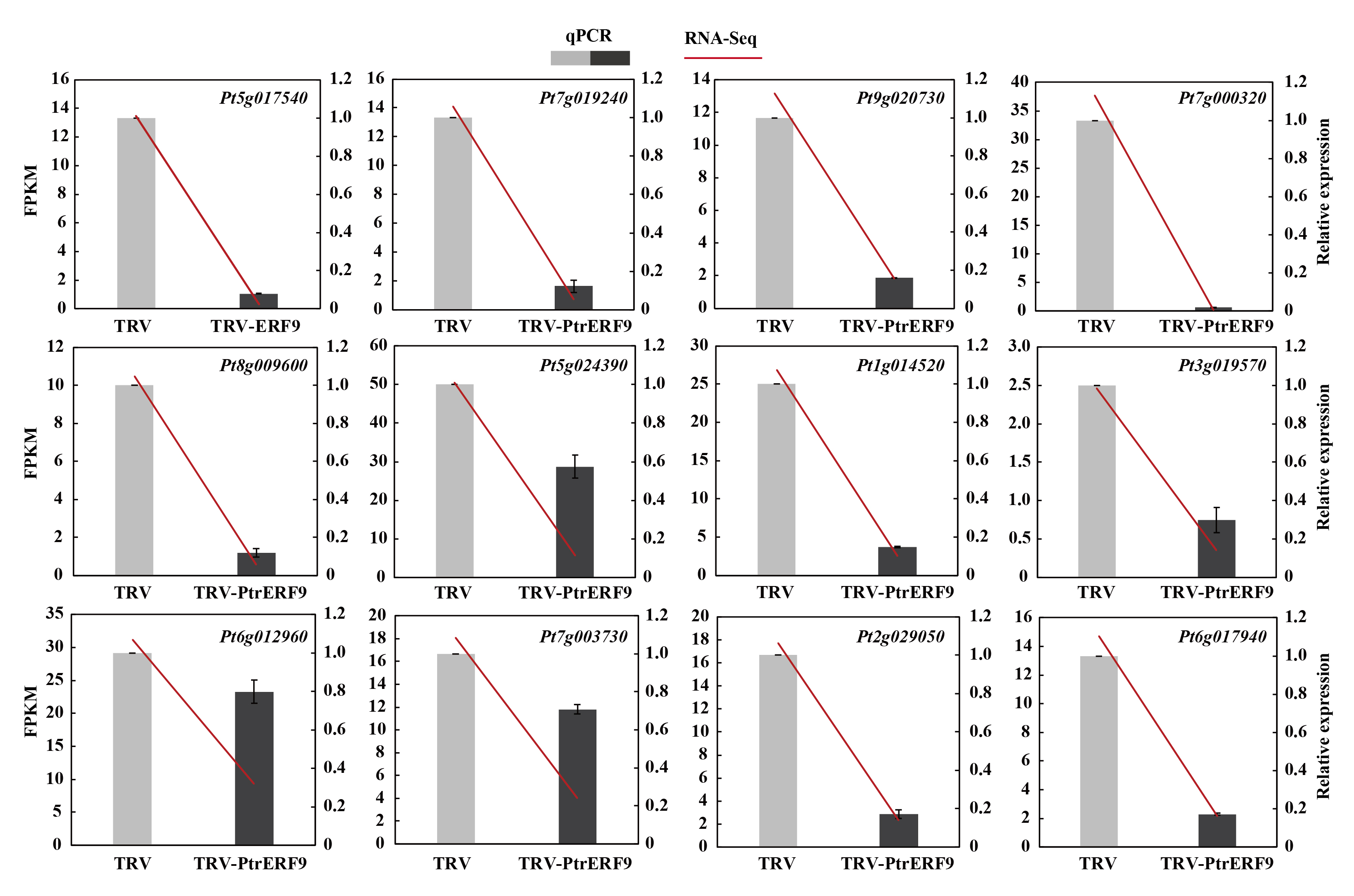


**Figure S6. Validation of differentially expressed genes by qPCR analysis.** Transcript levels of twelve randomly selected DEGs of TRV-*PtrERF9* plants. The Y-axis on the left shows corresponding expression data of RNA-Seq and qPCR are shown using red lines and histograms, respectively. *Actin* was used as an internal control. Error bars indicate ± SE (n = 3).


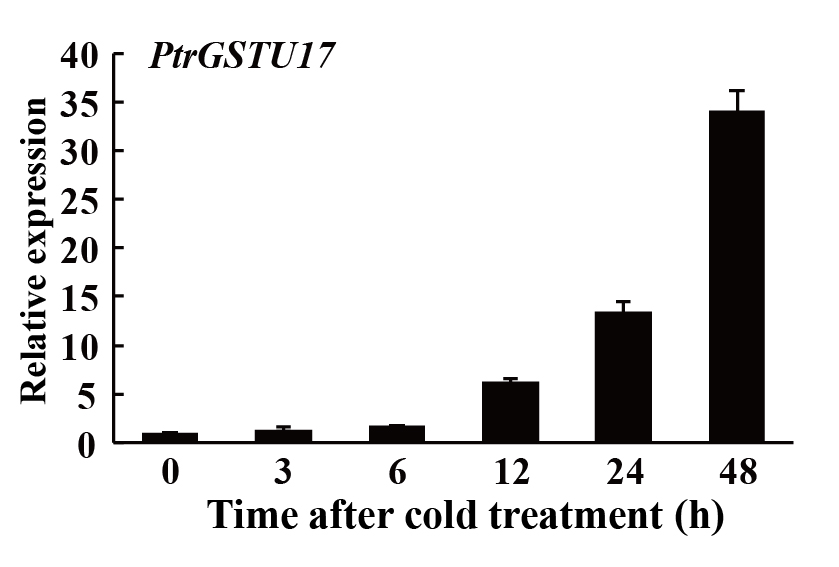


**Figure S7. Relative expression of *PtrGSTU17* at the designated time points of cold treatment by qPCR.** *Actin* gene was used as an internal control. Error bars indicate ± SE (n = 3).


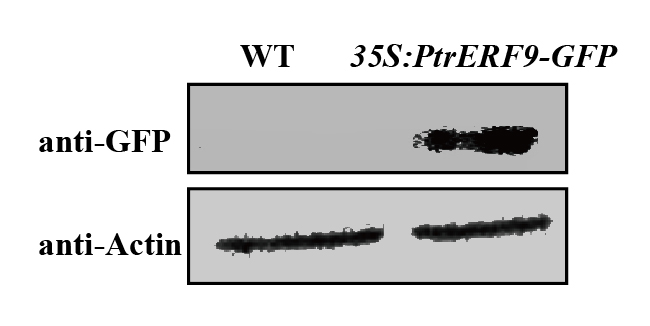


**Figure S8. Analysis of PtrERF9-GFP protein level in the *Poncirus trifoliata* leaves transiently expressing *PtrERF9* by western blot.** Actin protein was used as an internal control.


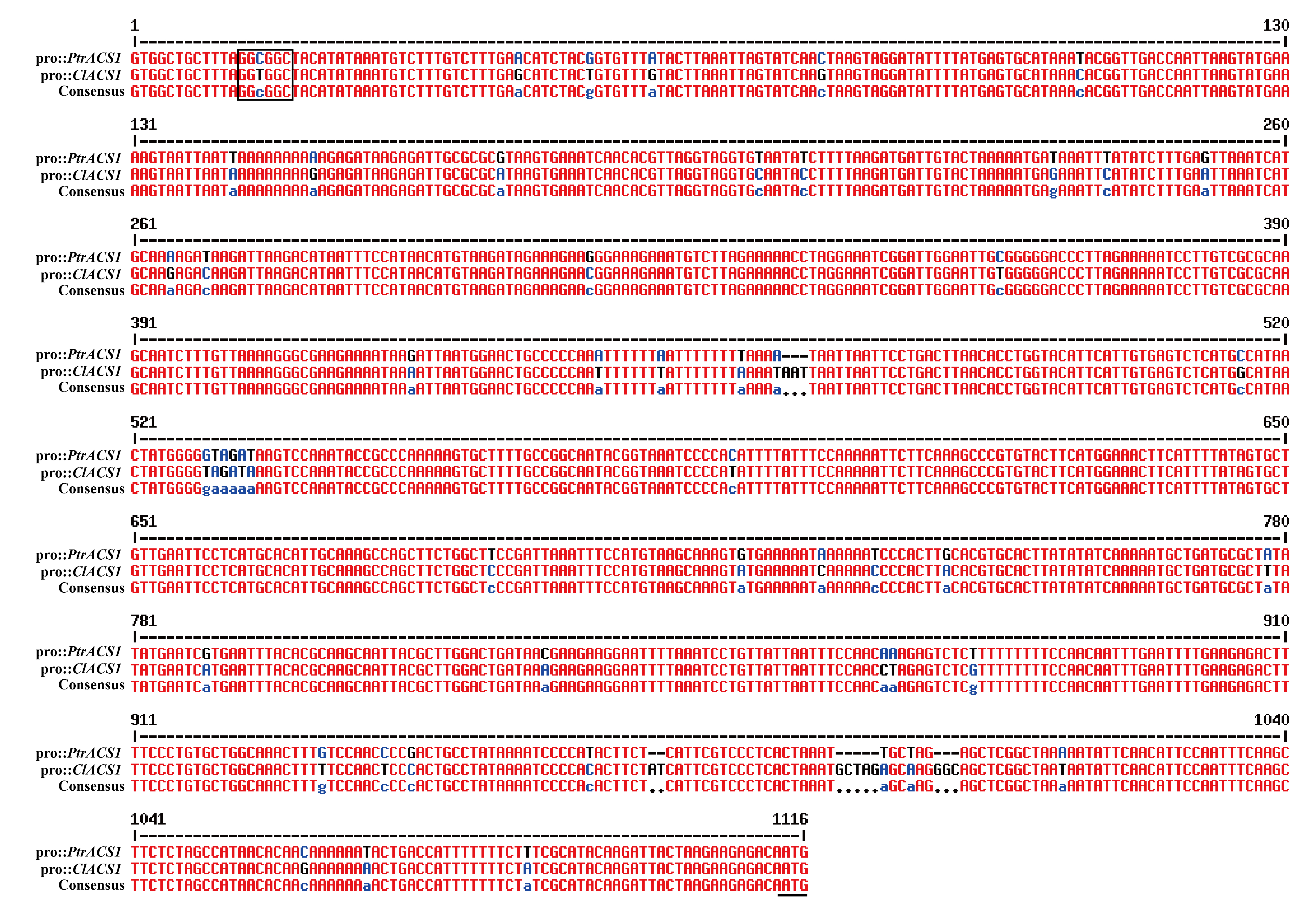


**Figure S9. Comparison and analysis of *PtrACS1* and *ClACS1* promoters.** The black box indicates the GCC-box of *PtrACS1* and mutated GCC-box of *ClACS1*. The black bar indicates start codon ATG.


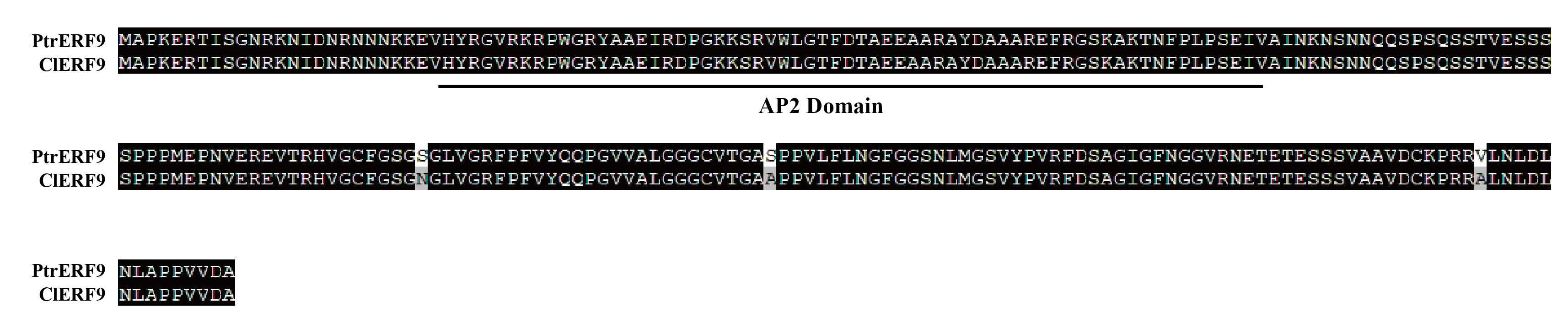


**Figure S10. Sequence alignments of PtrERF9 and ClERF9**. Identical amino acid residues are shown in black and gray shade. Black bar indicates AP2 domain.


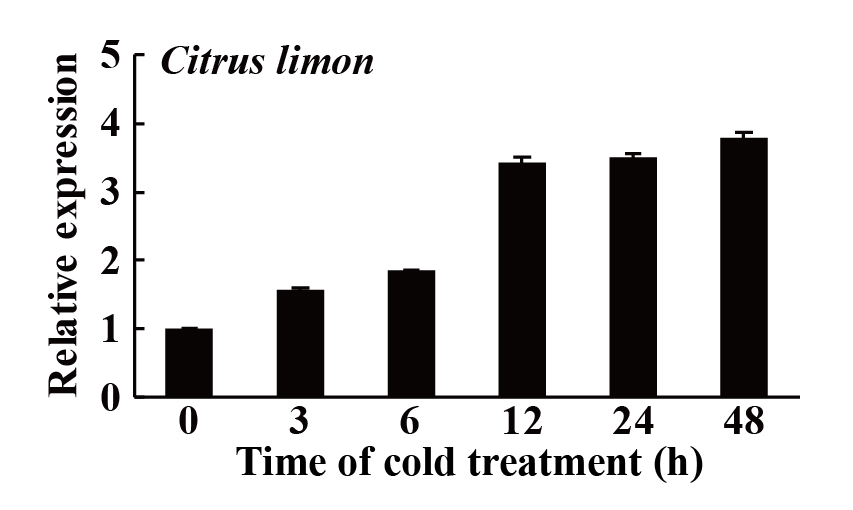


**Figure S11. Expression levels of *ClERF9* from *Citrus limon* under cold treatment.** *Actin* gene was used as an internal control. Error bars indicate ± SE (n = 3).

**Table S1.** List of primers used in this study.

| **Primers** | **Oligonucleotide sequences (5’ to 3’)** |
| --- | --- |
| **Gene expression and cloning** | |
| *Pt2g011090*-F | CTCTCCAAAACAAAAACAGCACACA |
| *Pt2g011090*-R | ACCGTATTAACCGGCTCATCAC |
| DX2181-pPtrERF9-F (*Pst* I) | CTACAGCGCTAAGCTTGGCTGCAGACTGTTTAGCAACTTTTAAGCAGC |
| DX2181-pPtrERF9-R (*Bam*HI) | AAGGGACTGACCACCCGGGATCCTGGTGCCATTGTTTTTTT |
| 101YFP-*PtrERF9*-F (*Eco*RI) | GGAATTCATGGCACCAAAAGAGAGAAC |
| 101YFP-*PtrERF9*-F (*Bam*HI) | CGGGATCCTGCGTCAACCACTGGTGG |
| pDONR222-*PtrERF9*-F | GGGGACAAGTTTGTACAAAAAAGCAGGCTTAATGGCACCAAAAGAGAGAAC |
| pDONR222-*PtrERF9*-R | GGGGACCACTTTGTACAAGAAAGCTGGGTTTGCGTCAACCACTGGTGG |
| *Ubiquitin-*F | GGTGTTTCCAGTGGCGGACG |
| *Ubiquitin-*R | TCCTCCCCTCAGCTACGGGGTAT |
| *PtrERF9-*F (qPCR) | CAGAGCAGTACCGTGGAGTC |
| *PtrERF9-*R (qPCR) | GTTCGATCCCCCAAACCCGT |
| *Actin*-F | CCGACCGTATGAGCAAGGAAA |
| *Actin*-R | TTCCTGTGGACAATGGATGGA |
| pTRV2-*PtrERF9*-F (*Bam*HI) | AGAAGGCCTCCATGGGGATCCGCCATCAACAAGAATAGC |
| pTRV2-*PtrERF9*-R (*Sma*I) | TGTCTTCGGGACATGCCCGGGGTTAAGAACCCTCCTTGG |
| *PtrGSTU17*-F (qPCR) | TCGAGTACGTGGATGAGGCT |
| *PtrGSTU17*-R (qPCR) | ATCAACGTAAGCGGCCCAG |
| *Pt3g019570-*F (qPCR) | GAAATGTCGGAGACGAGGGT |
| *Pt3g019570*-R (qPCR) | CACACTTGCCGTACAAGCTC |
| *Pt9g020730*-F (qPCR) | ATGCATGACCCGAAAGTCCC |
| *Pt9g020730*-R (qPCR) | AGCTTTGTTTTGCGGAGTCG |
| *Pt6g017940*-F (qPCR) | TCTTTCCGGTGGCAGGAATC |
| *Pt6g017940*-R (qPCR) | GCTGTTCAGCACTTTGGACG |
| *Pt7g019240*-F (qPCR) | GAGACTCCAAAGGTGCAAGC |
| *Pt7g019240*-R (qPCR) | GTGTGGCTCCCAGATAGTGC |
| *Pt8g009600*-F (qPCR) | ATGGCTACCATTGACACCGC |
| *Pt8g009600*-R (qPCR) | CCCCTGATGAAGCCATCGTC |
| *Pt2g029050*-F (qPCR) | AGTCACAGCCAGCAGCATTA |
| *Pt2g029050*-R (qPCR) | ATGAGCTCCAATGACACCCC |
| *Pt7g003730-*F (qPCR) | TGGCTAAACAAGCAAGCTCC |
| *Pt7g003730-*R (qPCR) | ACCAAACCAGGCCTAACCAC |
| *Pt6g012960-*F (qPCR) | GTTAGTGCTCCACTGAGGGTG |
| *Pt6g012960-*R (qPCR) | CAGCAGCAGCAGCCAAG |
| *Pt3g019570-*F (qPCR) | CGGTACGGCGAAAAGGAGTGG |
| *Pt3g019570-*R (qPCR) | CCAGCGTAGAACACAAGCGC |
| *Pt7g000320-F* (qPCR) | TATTGTCAGCAGGAACGCCA |
| *Pt7g000320-R* (qPCR) | AGCCAACATCGTTCCAGAGG |
| *Pt1g014520*-F (qPCR) | AGTATTGTGCCGGTGGTGAG |
| *Pt1g014520*-R (qPCR) | TCAGTGACCTTCAAACGAGCA |
| *Pt1g014520*-F (qPCR) | GTCATTGAGGCGTTTCGAGC |
| *Pt1g014520*-R (qPCR) | TGCACTGCAAACACAAGAGC |
| *PtrACS1*-F (qPCR) | TGCACTAAACCGAATTGCCG |
| *PtrACS1*-R (qPCR) | TGAGGAGACATCATGTGCGG |
| *Pt2g025450*-F (qPCR) | AGAAGGCTTCGGGACCTGTA |
| *Pt2g025450*-R (qPCR) | GGATCCCTAATCTCGGCTGC |
| *Pt1g010930*-F (qPCR) | AAACTTCCCAACGCCAAACG |
| *Pt1g010930*-R (qPCR) | AAACTTCCCAACGCCAAACG |
| *Pt2g025470*-F (qPCR) | TCAGCACCGTTGGATCACTC |
| *Pt2g025470*-R (qPCR) | TCAATAACCGCAGCGTCAGT |
| *Pt2g015700*-F (qPCR) | ACATCCGATACAGAGGCGTG |
| *Pt2g015700*-R (qPCR) | CTGAGTCGAAGGTGCCGAG |
| *Pt1g015160*-F (qPCR) | AGCACCGTCGAGTCATTCAG |
| *Pt1g015160*-R (qPCR) | ATGACAGTCCTCCGGAACCA |
| *Pt3g040700*-F (qPCR) | GGGAAAGTTTGCTGCGGAGA |
| *Pt3g040700*-R (qPCR) | CGCATCTTGAAAGCGGCTC |
| **Y1H assay** |  |
| pGADT7-*PtrERF9*-F (*Nde* I) | GATTACGCTCATATGATGGCACCAAAAGAGAGAAC |
| pGADT7-*PtrERF9*-R (*Eco*R I) | ACCCGGGTGGAATTCTCATGCGTCAACCACTGGTG |
| pGADT7-*ClERF9*-F (*Nde* I) | GATTACGCTCATATGATGGCACCAAAAGAGAGAAC |
| pGADT7-*ClERF9*-R (*Eco*R I) | ACCCGGGTGGAATTCTCATGCGTCAACCACTGGTG |
| pAbAi-*PtrGSTU17* (P1) -F (*Kpn* I) | CTTGAATTCGAGCTCGGTACCATTCCAACGAGCCGCCAGCTT |
| pAbAi-*PtrGSTU17* (P1) -R (*Xho* I) | ATACAGAGCACATGCCTCGAGCCGTAGCCATGTCCCATT |
| pAbAi-*PtrACS1* (P2)-F (*Kpn* I) | CTTGAATTCGAGCTCGGTACCAGGACGGTGGCTGCTTT |
| pAbAi-*PtrACS1* (P2)-R (*Xho* I) | ATACAGAGCACATGCCTCGAGGTTGATTTCACTTACGCGCGC |
| pAbAi-*ClACS1* (P3)-F (*Kpn* I) | CTTGAATTCGAGCTCGGTACCCGTTAGCCGGTAAAGGAC |
| pAbAi-*ClACS1* (P3)-R (*Xho* I) | ATACAGAGCACATGCCTCGAGGCTCTAGCAATTTAGTGAGGGACG |
| **EMSA** |  |
| P1- probe | CCATCCAATTCCAACGAGCCGCCAGCTTATAATAACAACA |
| mP1-probe | CCATCCAATTCCAACGATCCTCCAGCTTATAATAACAACA |
| P2- probe | AGGACGGTGGCTGCTTTAGGCGGCTACATATAAATGTCTT |
| mP2- probe | AGGACGGTGGCTGCTTTAGGTGGCTACATATAAATGTCTT |
| **LUC assay** |  |
| 62-SK-*PtrERF*-F (*Bam*H I) | CGCTCTAGAACTAGTGGATCCATGGCACCAAAAGAGAGAAC |
| 62-SK-*PtrERF9*-R (*Eco*R I) | GATAAGCTTGATATCGAATTCTCATGCGTCAACCACTGGTG |
| LUC-*PtrGSTU17*-0800-F (*Hind* III) | GTCGACGGTATCGATAAGCTTATTCCAACGAGCCGCCAGCTT |
| LUC-*PtrGSTU17*-0800-R (*Bam*H I) | CGCTCTAGAACTAGTGGATCCTTTTTTTTTTTCTCTAAGCTCTGC |
| LUC-*PtrACS1*-0800-F (*Hind* III) | GTCGACGGTATCGATAAGCTTGACGGTGGCTGCTTTAGGCGGC |
| LUC-*PtrACS1*-0800-R (*Bam*H I) | CGCTCTAGAACTAGTGGATCCGCCAGCACAGGGAAAAGTCTC |
| **ChIP-qPCR assay** |  |
| F1 fragment-F | GCTTTTGCTCTCCGGTTGAC |
| F1 fragment-R | CCGATGAGTTGCTTGGGTTC |
| F2 fragment-F | ACCAGTTACCGCCCTTGAGCC |
| F2 fragment-R | CTTGCAGCTTCTGCGCCGTAGCC |
| F3 fragment-F | GTGGCTGCTTTAGGCGGC |
| F3 fragment-R | GTTGATTTCACTTACGCGCGC |
| F4 fragment-F | TCATGCACATTGCAAAGCCAG |
| F4 fragment-R | AGTCCAAGCGTAATTGCTTGC |

**Table S2.** Summary of RNA-seq results.

| Sample | Total Raw Reads (M) | Total Clean Reads (M) | Total Clean Bases (Gb) | Clean Reads Q20 (%) | Uniquely mapping gene ratio |
| --- | --- | --- | --- | --- | --- |
| EV1 | 47.43 | 43.6 | 6.54 | 97.56 | 95.88% |
| EV2 | 47.43 | 43.72 | 6.56 | 97.41 | 95.84% |
| EV3 | 47.43 | 43.6 | 6.54 | 97.56 | 95.78% |
| E9-1 | 49.19 | 43.86 | 6.58 | 96.64 | 94.36% |
| E9-2 | 49.19 | 44.67 | 6.7 | 96.71 | 94.65% |
| E9-3 | 49.19 | 44.96 | 6.74 | 96.78 | 94.70% |

Note: “EV” TRV control plants, “E9” TRV-ERF9 VIGS plants.
